# Assessing the Accuracy and Completeness of AI-Generated Dental Responses: An Evaluation of the Chat-GPT Model

**DOI:** 10.3390/healthcare13172144

**Published:** 2025-08-28

**Authors:** Ahmad A. Othman, Abdulwadood J. Sharqawi, Ahmed A. MohammedAziz, Wafaa A. Ali, Amjad A. Alatiyyah, Mahir A. Mirah

**Affiliations:** 1 Department of Oral and Maxillofacial Diagnostic Sciences, College of Dentistry, Taibah University, Al-Madinah Al-Munawwarah 42353, Saudi Arabia; aaaothman@taibahu.edu.sa; 2 Department of Preventive Dental Sciences, College of Dentistry, Taibah University, Al-Madinah Al-Munawwarah 42353, Saudi Arabia; asharqawi@taibahu.edu.sa; 3 College of Dentistry, Taibah University, Al-Madinah Al-Munawwarah 42353, Saudi Arabia; dra7madaziz@gmail.com (A.A.M.); dr.wafaamurdan@gmail.com (W.A.A.); dr.alatiyyah@gmail.com (A.A.A.); 4 Department of Restorative Dental Sciences, College of Dentistry, Taibah University, Al-Madinah Al-Munawwarah 42353, Saudi Arabia

**Keywords:** health informatics, large language models, dentistry, educational technology, GPT-3.5, GPT-4

## Abstract

**Background:** The rapid advancement of artificial intelligence (AI) in healthcare has opened new opportunities, yet the clinical validation of AI tools in dentistry remains limited. **Objectives:** This study aimed to assess the performance of ChatGPT in generating accurate and complete responses to academic dental questions across multiple specialties, comparing the capabilities of GPT-4 and GPT-3.5 models. **Methodology:** A panel of academic specialists from eight dental specialties collaboratively developed 48 clinical questions, classified by consensus as easy, medium, or hard, and as requiring either binary (yes/no) or descriptive responses. Each question was sequentially entered into both GPT-4 and GPT-3.5 models, with instructions to provide guideline-based answers. The AI-generated responses were independently evaluated by the specialists for accuracy (6-point Likert scale) and completeness (3-point Likert scale). Descriptive and inferential statistics were applied, including Mann–Whitney U and Kruskal–Wallis tests, with significance set at *p* < 0.05. **Results:** GPT-4 consistently outperformed GPT-3.5 in both evaluation domains. The median accuracy score was 6.0 for GPT-4 and 5.0 for GPT-3.5 (*p* = 0.02), while the median completeness score was 3.0 for GPT-4 and 2.0 for GPT-3.5 (*p* < 0.001). GPT-4 demonstrated significantly higher overall accuracy (5.29 ± 1.1) and completeness (2.44 ± 0.71) compared to GPT-3.5 (4.5 ± 1.7 and 1.69 ± 0.62, respectively; *p* = 0.024 and <0.001). When stratified by specialty, notable improvements with GPT-4 were observed in Periodontology, Endodontics, Implantology, and Oral Surgery, particularly in completeness scores. **Conclusions:** In academic dental settings, GPT-4 provided more accurate and complete responses than GPT-3.5. Despite both models showing potential, their clinical application should remain supervised by human experts.

## 1. Introduction

Artificial intelligence (AI) alludes to the theory and application of computer systems that have the ability to perform duties that would usually require human intelligence [1]. AI has been more involved in healthcare and dentistry. This can increase the accuracy, efficiency, and effectiveness of patient care whilst minimizing the clinical workload and cost [2]. The AI is able to analyze and process large-scale data including cone-beam computed tomography, radiographs, clinical records, supporting diagnostic treatment planning, and making decisions [3]. In addition, AI can be used in detecting dental and craniofacial disorders, such as fractures, caries, and periodontal diseases, with improved diagnostic accuracy and time [3,4,5,6].

Within AI-used applications, the Chatbot Generative Pre-Trained Transformer (ChatGPT), developed by OpenAI in 2022, has drawn a lot of attention for its ability to provide natural language processing duties [7]. Able of generating humanized text based on specific input prompts, ChatGPT has been used in many different fields, including academic learning, healthcare, and scientific publishing [7]. In academic dentistry, artificial intelligence models have been found to help and assist dental researchers in having better literature writing and scientific development [7,8,9,10,11]. On the other hand, many concerns were found to be important regarding the accuracy and completeness of the information that is exerted from ChatGPT, specifically when it is used as a valid source in the health sector [12]. The AI-responses may not have all the clinical depth because they may provide partial answers, which could limit some of the educational component. In dentistry, limited evidence exists regarding the completeness and accuracy of AI-generated dental responses [12,13]. At the same time, academic dentists increasingly have explored ChatGPT’s role in providing better academic writing, translation, and theoretical development, without excluding the limitations that could be found in the context’s reliability and appropriateness [14,15]. Even with the availability of developed versions such as GPT-4, performance quality and unconfronted concerns about its consistency across dental specialties and clinical topics remain evident [16,17].

Considering the important role of accurate, complete, and clinically reliable information in dental education and practice, we should take time to assess the performance of AI-based tools. In this regard, this study aimed to assess the accuracy and completeness of responses generated by ChatGPT 3.5 and to compare its performance with the more developed version GPT-4 model. Together, these metrics reflect both the correctness and the clinical relevance of AI-generated answers. They took the questions of experts as a benchmark, and the assessment was performed on various dental specialties. Likert scales were chosen for their simplicity and comparability in structuring expert judgments, though their inherent subjectivity was mitigated by using multiple independent assessors.

## 2. Materials and Methods

### 2.1. Study Design and Question Development

A cross-sectional, expert panel–based comparative evaluation study designed to assess the performance of ChatGPT (versions 3.5 and 4) across eight dental specialties. Academic specialties with following disciplines: Orthodontics, Endodontics, Implantology, Pediatric Dentistry, Periodontology, Preventive Dentistry, Prosthodontics, and Oral Surgery, formulated a set of 48 questions, which they subjectively rated as easy, medium, or hard, requiring either binary (yes/no) or descriptive responses. All eight specialists, each with more than five years of teaching and clinical experience, were briefed on the scoring criteria and calibrated with examples to ensure consistency. The ChatGPT 3.5 and 4 bots were used to answer the questions. Specific instructions were provided to give answers that adhere to the guidelines. A single investigator facilitated this process. The specialists evaluated the responses from ChatGPT based on accuracy, using a 6-point Likert scale (1 for ‘completely incorrect’ to 6 for ‘completely correct’), and completeness, employing a 3-point Likert scale (1 for ‘incomplete’ to 3 for ‘complete with additional context’). Completeness refers to the extent to which responses address all relevant subpoints, align with established guidelines, and present a coherent answer structure. The scores were collected and analyzed using descriptive statistics to compare the performance of and between the two ChatGPT versions.

### 2.2. Data Collection Procedure

After the questions were collected, each of the 48 questions was serially entered into ChatGPT, utilizing both Generative Pre-Trained Transformer, version 4 (GPT-4) and Generative Pre-Trained Transformer, version 3.5 (GPT-3.5) models. Instructions were given to ChatGPT to give comprehensive, guideline-based clinical answers consistent with current dental practice standards. All the questions were typed into the chatbot of both models by one investigator with the same instructions and settings, to guarantee the consistency of the procedure and reduce variability. The obtained answers provided by AI were then anonymized and systematically compiled for independent evaluation. The study protocol was reviewed and approved by Taibah University Institutional Review Board. Moreover, AI in education should balance innovation with integrity, minimize bias, and support learners’ critical thinking and independence.

### 2.3. Response Evaluation

Each of the two versions of GPT was reviewed and scored separately by the same eight academic dental specialists who formulated the original questions. The specialists evaluated the responses that concerned their area of expertise, and the evaluations were content-appropriate and informed. The responses generated by AI were evaluated in two evaluation domains. Accuracy was measured using a 6-point Likert scale, where a score of 1 denoted a completely incorrect response and a score of 6 indicated a completely correct answer that adhered to established clinical guidelines. In parallel, completeness was evaluated using a 3-point Likert scale, with 1 representing an incomplete response, 2 indicating a sufficiently complete answer that adequately addressed the question, and 3 reflecting a fully complete response supplemented with additional relevant clinical context or explanation. All the assessments were documented in an organized dataset and prepared for subsequent statistical analysis.

### 2.4. Statistical Analysis

Descriptive analyses were used for both versions of ChatGPT to analyze medians, means, and standard deviation for accuracy and completeness scores. These were summed up the overall and further divided by difficulty of the question (easy, medium, hard), type of question (binary, descriptive), and dental specialty. The Shapiro–Wilk test was used to analyze if the data were not normally distributed. Consequently, Mann–Whitney U test was used to compare the mean accuracy and completeness scores of the two versions of GPT, whereas Kruskal–Wallis test was used to compare the scores of multiple groups. The statistical tests were all two-tailed and a *p*-value < 0.05 was regarded as statistically significant. Statistical Package for the Social Sciences (SPSS) software version 22 was used to analyze data.

## 3. Results

Both GPT-4 and GPT-3.5 models answered 48 clinical dental questions, providing 96 AI responses. Eight dental academic experts assessed these answers based on the set criteria of accuracy and completeness. The accuracy and completeness scores of AI-generated answers to the study questions are provided in Table 1.

The scores of accuracy and completeness of ChatGPT answers were presented in Table 1. In the Multispecialty group (*n* = 48), the overall mean accuracy was 9.8 ± 2.4 with a median of 11.0, while the mean completeness score was 4.1 ± 1.1 with a median of 4.0. Accuracy scores in this group were highest for easy questions (10.9 ± 1.4/11.0) and lowest for hard questions (9.2 ± 2.6/10.0). Completeness scores followed a similar pattern, ranging from 4.5 ± 0.94/5.0 in easy questions to 3.80 ± 0.83/4.0 in hard questions. Orthodontics was one of the individual specialties with consistent mean accuracy scores of 12.00 ± 0.00 with a median of 12.0 across all difficulty levels. Completeness scores in this specialty varied slightly, with the highest for easy questions (5.00 ± 0.00/5.0) and the lowest for medium difficulty (3.50 ± 2.12/3.5). Pediatric Dentistry was also highly accurate, with means ranging from 11.00 ± 1.26/11.5 to 9.50 ± 0.71/9.5, and completeness scores consistently more than 4.5.

In contrast, Preventive Dentistry has lower overall accuracy with a mean of 8.17 ± 1.34/8.0, ranging from 9.00 ± 1.00/9.0 in easy questions and in medium difficulty was 7.50 ± 2.12/7.5. Completeness scores in this specialty ranged from 3.50 ± 0.71/3.5 to 3.00 ± 0.0/3.0. The answers to Oral Surgery questions had high accuracy scores, with overall means of 11.08 ± 1.88/11.5, peaking at 12.00 ± 0.0/12.0 in hard questions, while completeness scores ranged from 5.00 ± 0.00/5.0 in easy questions to 4.50 ± 0.71/4.5 in hard questions. The answers to Implantology questions showed variable accuracy scores, highest in medium difficulty (12.00 ± 0.00/12.0) and lowest in hard questions (7.50 ± 4.95/7.5), with completeness scores similarly ranging from 5.5 ± 0.00/5.0 to 3.00 ± 1.41/3.0.

The accuracy scores of other specialties like Endodontics, Periodontology, Prosthodontics, and Multispecialty were between 6.50 and 11.5, and completeness scores were between 2.5 and 5.0 depending on the type and difficulty of the questions. In all specialties, the scores of accuracy and completeness were higher in easier questions and lower in harder ones, and the standard deviations and medians varied, indicating the differences in the consistency of performance.

In Table 2, the accuracy score of GPT-4 and GPT-3.5 is shown according to the types and difficulty of the question. For both question types combined, the overall mean accuracy score for GPT-4 was 5.29 ± 1.1 with a median of 6.0, compared to 4.5 ± 1.7 and a median of 5.0 for GPT-3.5, with a statistically significant difference (*p* = 0.024).

In the binary questions, the average accuracy scores of GPT-4 were 4.50 ± 1.60 in hard questions to 5.88 ± 0.35 in easy questions, whereas the average accuracy scores of GPT-3.5 were 3.75 ± 2.25 to 5.38 ± 1.4 in hard and easy questions, respectively. None of the *p*-values for binary questions reached statistical significance.

For descriptive questions, GPT-4 mean accuracy scores ranged from 5.13 ± 0.99 in medium difficulty to 5.75 ± 0.46 in easy questions, while GPT-3.5 scores ranged from 4.75 ± 1.16 to 5.33 ± 1.58. Although statistically significant differences were not observed in descriptive questions across difficulty levels, a borderline significant difference was observed for all questions (*p* = 0.049). Median score of accuracy of both models was found to be between 4.0 and 6.0 in all categories.

In the binary questions, the mean accuracy scores of GPT-4 were 4.50 ± 1.60 in hard questions to 5.88 ± 0.35 in easy questions, whereas GPT-3.5 scores were 3.75 ± 2.25 to 5.38 ± 1.4 in hard and easy questions, respectively. All the *p*-values of binary questions were not statistical significance.

In descriptive questions, the mean accuracy scores of GPT-4 were 5.13 ± 0.99 in medium difficulty and 5.75 ± 0.46 in easy questions, whereas GPT-3.5 scores were 4.75 ± 1.16 to 5.33 ± 1.58. Although statistically significant differences were not observed in descriptive questions across difficulty levels, a borderline significant difference was observed for all questions (*p* = 0.049). The median accuracy scores of the two models were mostly between 4.0 and 6.0 in all categories.

Table 3 shows the completeness scores of GPT-4 and GPT-3.5 based on the type of question and the level of difficulty. The overall mean of completeness score of GPT-4 of both types of questions was 2.44 ± 0.71, with a median of 3.0, whereas the corresponding values of GPT-3.5 were 1.69 ± 0.62 and 2.0, respectively, with a statistically significant difference (*p* < 0.0001). The mean of completeness scores of GPT-4 were 2.75 ± 0.46 and 2.13 ± 0.83 in easy and hard questions, respectively, as compared to 1.88 ± 0.64 and 1.63 ± 0.74 in the same difficulty levels with GPT-3.5. Although a significant difference was observed for all binary questions (*p* = 0.001), the significant differences were observed for only easy (*p* = 0.011) and medium (*p* = 0.028) difficulty levels, but not for hard questions (*p* = 0.219).

**Figure 1:**
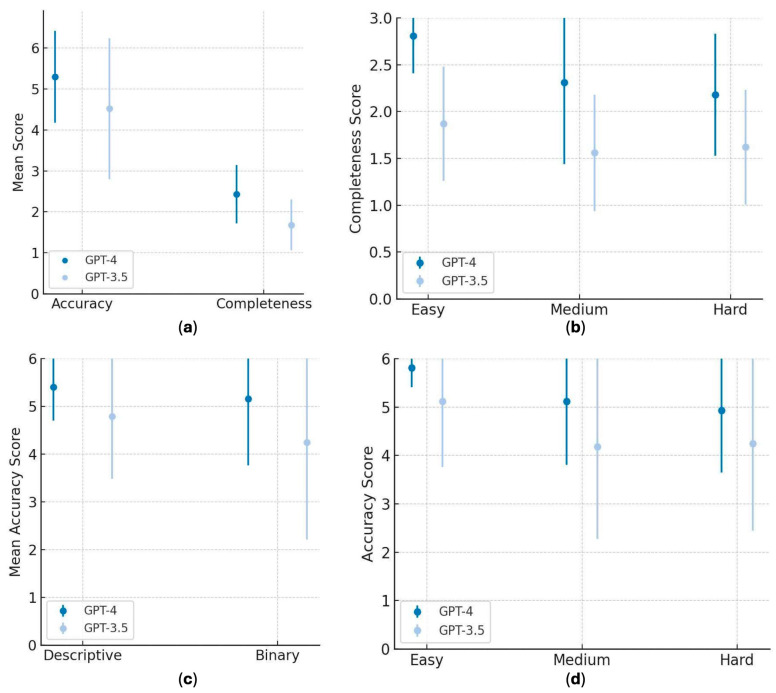
(**a**) Comparative analysis of mean accuracy and completeness scores: GPT-4 vs. GPT-3.5. (**b**) Mean completeness scores of GPT-4 vs. GPT-3.5 across question difficulty levels. (**c**) Mean accuracy scores of GPT-4 vs. GPT 3.5 for binary and descriptive types of questions. (**d**) Mean accuracy scores of GPT-4 vs. GPT-3.5 across question difficulty levels.

In descriptive questions, the mean completeness scores of GPT-4 ranged between 2.88 ± 0.35 and 2.25 ± 0.46 in easy and hard questions, respectively. GPT-3.5, however, had mean scores of 1.88 ± 0.64 in the easy questions and 1.63 ± 0.51 in hard questions. Statistically significant differences were noted for all descriptive questions (*p* < 0.0001), particularly at the easy (*p* = 0.004) and hard (*p* = 0.029) levels, while no significant difference was found for medium difficulty descriptive questions (*p* = 0.221). GPT-4 median of completeness scores were always between 2.0 and 3.0, whereas GPT-3.5 medians were between 1.0 and 2.0 in all categories. Figure 1 illustrates the results of Table 2 and Table 3, the mean accuracy and completeness scores of GPT-4 and GPT-3.5 on the studied questions, by type and difficulty level of the question.

Table 4 shows a comparative summary of the performance metrics of GPT-4 and GPT-3.5 when compared in overall categories, dental specialties, question types, and difficulty levels. In the overall category, GPT-4 achieved a mean accuracy score of 5.29 ± 1.1 with a median of 6.0, while GPT-3.5 recorded 4.5 ± 1.7 with a median of 5.0; the associated *p*-value was 0.024. For completeness, GPT-4 attained a mean score of 2.44 ± 0.71 with a median of 3.0, compared to 1.69 ± 0.62 for GPT-3.5, with a *p*-value of <0.001.

In specialty-based analysis, accuracy scores ranged from 4.30 ± 1.60 in Prosthodontics and 6.00 ± 0.00 in Implantology and Oral Surgery for GPT-4. Completeness scores for GPT-4 varied between 2.17 ± 0.75 in Preventive Dentistry and 2.67 ± 0.81 in Pediatric Dentistry and Oral Surgery. Notable *p*-values for accuracy included 0.001 in Periodontology and for completeness, significant differences were observed in Endodontics (0.04), Implantology (0.04), Periodontology (0.01), Preventive Dentistry (0.02), and Oral Surgery (0.02).

The two models showed the same mean accuracy scores in Implantology (6.00 ± 0.00) and Pediatric Dentistry (5.50 ± 1.22 for GPT-4 vs. 5.50 ± 0.84 for GPT-3.5). Completeness scores showed minimal variation in Pediatric Dentistry (2.67 ± 0.81 vs. 2.33 ± 0.81) with a non-significant *p*-value of 0.33.

The questions and answers generated from ChatGPT 3.5 and 4 are presented in Table A1, Table A2, Table A3, Table A4, Table A5, Table A6, Table A7 and Table A8, which are available in Appendix A.

## 4. Discussion

The use of AI is growing fast in health education and research [18], with growing applications in dental medicine as well. This study identified impressive differences in the accuracy and completeness of AI-generated dental responses across dental specialties and the difficulty of questions. Among the specialties, Pediatric Dentistry and Orthodontics were consistently ranked higher than others, which could be a sign of awareness in AI of particular clinical materials. Also, the accuracy and completeness scores tended to decrease with the increasing difficulty of the questions. For example, in the Multispecialty group, accuracy decreased by 10.9 ± 1.4 to 9.2 ± 2.6 for easy and hard questions, respectively, with a similar reduction in completeness. These findings are consistent with the existing studies that show that the more complex the duties are, the lower the AI performance in healthcare contexts, and recent evidence supports this pattern. As an example, Yang et al. [19] discovered that GPT-4 performed better on United States Medical Licensing Examination (USMLE) questions than previous models, but its performance and quality of answers decreased with the complexity of the question. Liu [20] found that GPT-4 was less accurate on multilingual medical licensing exams as clinical case complexity increased, and there was considerable subspecialty variation. Also, Ayan et al. [18] showed that students trained with the help of AI achieved promising results in the caries lesion detection, which indicates the possible use of AI in enhancing clinical education of dental students.

Additionally, the better performance of Orthodontics and Pediatric Dentistry can be linked to the nature of content of these two different specialties, where structured, protocol-based questions can be more aligned with the language model training data, an idea that was previously observed in the study, which found better AI output in more standardized, evidence-based medical fields [21].

The results of the study show that GPT-4 was much more accurate, in general, than GPT-3.5 when tested on both types of questions combined, with a mean of 5.29 ± 1.1 versus 4.5 ± 1.7, respectively (*p* = 0.024). This result indicates GPT-4’s improved capability in performing medical question-answering tasks. In the case of binary questions, both models showed the best performance on easy items and the worst on hard ones, but the differences were non-significant (*p*-values ranging from 0.163 to 0.354). The same trend was observed with descriptive questions, where GPT-4 performed better than GPT-3.5 at all difficulty levels, but not statistical significance (*p*-values between 0.182 and 0.542). These findings are consistent with the recent studies that compared GPT models in medical and academic settings. A recent study has shown that GPT-4 performed better than GPT-3.5 on questions on dentist board-style exams, particularly in questions that involved recall and interpretation. While GPT-4 also demonstrated an improvement in problem-solving tasks, these differences were less significant and not in all cases statistically significant, reflecting the findings of other subgroup [22]. In the same way, a study conducted by Rosol et al. [23] reported that GPT-4 performed better than GPT-3.5 in all three medical tests in both Polish and English. GPT-4 achieved a mean accuracy of 79.7% in both languages, passing all versions of the Medical Final Examination. On the other hand, GPT-3.5 attained mean accuracies of 54.8% in Polish and 60.3% in English, failing most of the Polish versions but passing all English versions regardless of temperature settings. Overall, these findings indicate that although GPT-4 showed significant improvements in accuracy compared to GPT-3.5, the level of its advantage may show some difference based on task type and difficulty. Identifying more subtle differences between the two versions might need larger sample size or more sensitive evaluation procedures [23].

The study results showed that GPT-4 consistently outperformed GPT-3.5 in completeness scores across various question types and difficulty levels. In general, GPT-4 achieved a much higher mean completeness score (2.44 ± 0.71) than GPT-3.5 (1.69 ± 0.62, *p* < 0.0001), indicating that it is more capable of giving comprehensive medical answers. GPT-4 was better than GPT-3.5 in almost every category by question type and difficulty. In binary questions, significant differences appeared at the easy (*p* = 0.011) and medium (*p* = 0.028) levels, while the difference in hard questions was not statistically significant (*p* = 0.219). In descriptive questions, GPT-4 had much better scores in easy (*p* = 0.004) and hard (*p* = 0.029) categories, with no significant difference at the medium level (*p* = 0.221). In both models, the completeness scores were higher on easier questions and lower on harder ones, which is consistent with other AI performance evaluations in which task difficulty affects the completeness of content. Similarly to other studies, the present study found that ChatGPT performed better on easier questions, particularly in oral medicine and dentistry, and accuracy and confidence decreased with the difficulty of the question. This trend was especially pronounced when it came to questions that necessitated recent research or advanced clinical judgment, which is known to be a weakness of AI systems in the management of complex cases [16,23]. These trends have been observed in other healthcare AI assessments. According to Takagi et al., GPT-3.5 scored 33.3% on challenging questions, whereas GPT-4 scored 40%, which is 17% higher than human examinees [24]. Similarly, Luo et al. [22] showed that GPT-4 was superior to GPT-3.5 regarding response completeness and relevance, especially, in easier and moderately difficult specialty board-style questions. The recent benchmarking explains the higher performance of GPT-4 by the increased token window and the enhanced reasoning capabilities that help to provide more comprehensive answers to open-ended questions. However, the models’ discrepancies are narrowed on challenging items, probably because of the intrinsic difficulty of AI to understand highly complex clinical reasoning tasks; a limitation that is reported in multiple studies [19,20,23].

In terms of accuracy and completeness of ChatGPT-4 and GPT-3.5 in different dental specialties, this study indicated that GPT-4 outperformed GPT-3.5 in Periodontology, Oral Surgery, Endodontics, and Implantology, with the most significant increase being completeness. These findings also indicate the increased ability of GPT-4 to work with complicated clinical material and produce more comprehensive, context-sensitive answers than previous versions. AI has also been used in dentistry, including diagnosis, detection of oral malignancies on radiographs, and evaluation of restorations [25], and in some cases, AI problem-solving skills can even outperform humans [26]. Recent cross-sectional analytical study of 70 dental cases in seven specialties compared the answers of ChatGPT-3.5 and GPT-4 evaluated by expert panels. GPT-4 was much more successful than GPT-3.5 in the overall quality of responses (67.1% vs. 44.3% rated as good; *p* = 0.016), especially in oral and maxillofacial surgery and complex cases, which indicates its improved contextual reasoning capabilities [27]. However, while both versions of ChatGPT, especially GPT-4, demonstrated good overall accuracy and excellent completeness in this study, their performance declined when addressing difficult questions. GPT-4, despite being overall better than GPT-3.5, was not able to provide fully comprehensive and contextually appropriate answers to hard questions most of the time. These were the more complicated cases that usually needed sophisticated clinical reasoning, subtle interpretation of new evidence, or specialized knowledge, in which the AI models were found to be weak. This implies that although AI-based tools such as ChatGPT have potential in assisting clinical education and decision-making, they might not be deep enough and capable of critical thinking to handle highly complex, specialized clinical cases [28]. In contrast to our structured, specialty-based evaluation of accuracy and completeness, Babaee Hemmati et al. [27] assessed GPT models using open-ended clinical scenarios and found GPT-4 superior, particularly in complex cases. While their design emphasizes clinical reasoning in realistic contexts, our study provides controlled benchmarking across eight specialties. Together, these complementary approaches underscore the need for both structured and scenario-based assessments to fully capture LLM performance in dentistry. A recent work emphasizes that by adopting a hybrid approach and advancing research in this area, the academic community can leverage AI’s efficiency while safeguarding the rigorous standards of scholarly inquiry that drive transformative discovery [29].

This study can provide important insights about the comparison performance of advanced large language models in the dental field, allowing one of the first systematic evaluations of GPT-4 and GPT-3.5 across multiple dental Desplaines, question types, and difficulty levels. The key strength of the study is its comprehensive design, including a large number of dental specialties and standardized question formats, which would be capable of increasing the reliability and clinical relevance of the findings. The scales of accuracy and completeness included allow for a comprehensive evaluation of the quality of the responses produced by AI, in terms of their factual correctness and the depth of the responses in the context of clinical decision support. Furthermore, this study employed a Likert scale to determine the accuracy and completeness of the responses provided by ChatGPT-3.5, which provides a systematic, consistent, and universally accepted approach to a profound and comprehensive assessment of AI performance in dental questions [30,31].

On the other hand, acknowledgment of the limitations is important. The question sample size per specialty was small, which may have led to the limitation of statistical power in subgroup analyses, specifically, in the case of more difficult questions, where the performance difference was lower. For free accessibility, availability and widespread use at the time of publication, ChatGPT-3.5 and 4.0 were selected for this study. Future studies should include other AI tools such as Gemini, Claude, or Bing Copilot to provide a broader comparative perspective. Potential sources of bias should be acknowledged in the interpretation of this study. First, assessor subjectivity may have influenced results, as individual experts could interpret Likert scale points differently despite prior calibration. Second, question design, although developed collaboratively, may have varied in complexity, scope, or adherence to guideline-based standards. Finally, the relatively small number of questions per specialty may have limited the representativeness of the findings. These factors highlight the need for larger, standardized question banks and broader assessor panels in future research to reduce variability and enhance generalizability. These limitations may have led to under- or overestimation of the true performance gap between GPT-4 and GPT-3.5. Larger, multicenter studies with broader and standardized question sets would strengthen generalizability and reduce bias. The reliance on a convenience sample in this study poses a potential limitation to the generalizability of the findings. Finally, AI and LLMs may generate biased or hallucinated content, and their role as learning “co-pilots” should be carefully managed to support education without fostering over-reliance.

## 5. Conclusions

In conclusion, the results of the current study show that GPT-4 is much more effective than GPT-3.5 in providing correct and complete dental responses, especially in such specialties as Periodontology, Preventive Dentistry, and Oral Surgery. It is suggested that future studies using bigger, real-world data, adopting a hybrid approach, and qualitative expert reviews would help to further confirm these findings and investigate how AI models can be practically implemented in the dental practice. Constant comparison with professional knowledge is still necessary to promote clinical safety, reliability, and educational worthiness in the context of adopting conversational AI in healthcare systems.

## Figures and Tables

**Table 1:** Accuracy and completeness scores for AI-generated dental answers.

Specialty	Both Question Type	Question Difficulty		
		Easy	Medium	Hard
	Accuracy Completeness Mean ± SD/Median	Accuracy Completeness Mean ± SD/Median	Accuracy Completeness Mean ± SD/Median	Accuracy Completeness Mean ± SD/Median
Multispecialty	9.8 ± 2.4/11.0 4.1 ± 1.1/4	10.9 ± 1.4/11.0 4.5 ± 0.94/5.0	9.3 ± 2.8/10.5 3.9 ± 1.3/4.0	9.2 ± 2.6/10.0 3.80 ± 0.83/4.0
Endodontics	9.50 ± 1.87/9.5 4.0 ± 1.3/3.5	10.50 ± 2.12/10.5 5.0 ± 0.71/4.5	9.00 ± 2.82/9.0 4.00 ± 0.0/4.0	9.00 ± 1.41/9.0 3.5 ± 0.70/3.5
Implantology	10.33 ± 2.98/11.5 4.30 ± 1.03/5.0	11.67 ± 0.58/11.5 5.5 ± 0.00/5.0	12.00 ± 0.0/12.0 5.00 ± 0.0/5.0	7.50 ± 4.95/7.5 3.00 ± 1.41/3.0
Orthodontics	9.50 ± 1.87/9.5 4.0 ± 1.3/3.5	12.00 ± 0.00/12.0 5.00 ± 0.00/5.0	12.00 ± 0.0/12.0 3.50 ± 2.12/3.5	12.00 ± 0.0/12.0 4.50 ± 0.72/4.5
Pediatric Dentistry	10.33 ± 2.98/11.5 4.30 ± 1.03/5.0	12.00 ± 0.00/12.0 5.50 ± 0.71/5.5	11.50 ± 0.7/11.5 5.00 ± 0.00/5.0	9.50 ± 0.71/9.5 4.50 ± 0.70/4.5
Periodontology	9.50 ± 1.87/9.5 4.0 ± 1.3/3.5	9.50 ± 1.00/9.5 4.00 ± 0.00/4.0	7.00 ± 1.41/7.0 3.50 ± 0.71/3.5	8.50 ± 2.12/8.5 4.00 ± 0.0/4.0
Preventive Dentistry	10.33 ± 2.98/11.5 4.30 ± 1.03/5.0	9.00 ± 1.00/9.0 3.50 ± 0.71/3.5	7.50 ± 2.12/7.5 3.50 ± 0.71/3.5	8.00 ± 0.0/8.0 3.00 ± 0.0/3.0
Prosthodontics	9.50 ± 1.87/9.5 4.0 ± 1.3/3.5	11.50 ± 0.71/11.5 5.00 ± 0.00/5.0	6.50 ± 4.94/6.5 2.50 ± 0.71/2.5	7.00 ± 4.24/7.0 3.50 ± 0.71/3.5
Oral Surgery	10.33 ± 2.98/11.5 4.30 ± 1.03/5.0	11.75 ± 0.96/11.5 5.00 ± 0.00/5.0	9.00 ± 2.82/9.0 4.00 ± 0.00/4.0	12.00 ± 0.0/12.0 4.50 ± 0.71/4.5

**Table 2:** Mean accuracy scores for GPT-4 vs. GPT-3.5 by question type and difficulty.

Question Type	Difficulty	GPT-4 Mean ± SD/Median	GPT 3.5 Mean ± SD/Median	*p*-Value
Both Types	All (*n* = 48)	5.29 ± 1.1/6.0	4.5 ± 1.7/5.0	0.024
Binary	All (*n* = 24)	5.17 ± 1.40/6.0	4.25 ± 2.04/5.0	0.077
	Easy (*n* = 8)	5.88 ± 0.35/6.0	5.38 ± 0.1.4/6.0	0.239
	Medium (*n* = 8)	5.13 ± 1.60/6.0	4.60 ± 2.1/4.0	0.163
	Hard (*n* = 8)	4.50 ± 1.60/5.0	3.75 ± 2.25/4.0	0.354
Descriptive	All (*n* = 24)	5.42 ± 0.77/6.0	4.79 ± 1.32/5.0	0.049
	Easy (*n* = 8)	5.75 ± 0.46/6.0	4.88 ± 1.35/5.0	0.182
	Medium (*n* = 8)	5.13 ± 0.99/5.5	5.33 ± 1.58/5.5	0.542
	Hard (*n* = 8)	5.38 ± 0.52/5.5	4.75 ± 1.16/4.5	0.371

**Table 3:** Mean completeness scores for GPT-4 vs. GPT-3.5 by question type and difficulty.

Question Type	Difficulty	GPT-4 Mean ± SD/Median	GPT 3.5 Mean ± SD/Median	*p*-Value
Both Types	All (*n* = 48)	2.44 ± 0.71/3.0	1.69 ± 0.62/2.0	<0.0001
Binary	All (*n* = 24)	2.42 ± 0.77/3.0	1.63 ± 0.64/2.0	0.001
	Easy (*n* = 8)	2.75 ± 0.46/3.0	1.88 ± 0.64/2.0	0.011
	Medium (*n* = 8)	2.38 ± 0.92/3.0	1.38 ± 0.51/1.0	0.028
	Hard (*n* = 8)	2.13 ± 0.83/2.0	1.63 ± 0.74/1.5	0.219
Descriptive	All (*n* = 24)	2.46 ± 0.65/3.0	1.75 ± 0.61/2.0	<0.0001
	Easy (*n* = 8)	2.88 ± 0.35/3.0	1.88 ± 0.64/2.0	0.004
	Medium (*n* = 8)	2.25 ± 0.88/2.5	1.75 ± 0.70/2.0	0.221
	Hard (*n* = 8)	2.25 ± 0.46/2.0	1.63 ± 0.51/2.0	0.029

**Table 4:** Comparison of GPT-4 and GPT-3.5 performance across specialties, question types, and difficulty levels.

Category	GPT-4 Accuracy Mean ± SD	GPT-3.5 Accuracy Mean ± SD	*p*-Value	GPT-4 Completeness Mean ± SD	GPT-3.5 Completeness Mean ± SD	*p*-Value
Orthodontics	5.00 ± 0.89	4.50 ± 1.05	0.86	2.3 ± 0.51	1.70 ± 0.81	0.11
Endodontics	5.17 ± 1.60	5.00 ± 1.60	0.34	2.50 ± 0.83	1.83 ± 0.41	0.04
Implantology	6.00 ± 0.00	6.00 ± 0.00	1.00	2.50 ± 0.83	1.83 ± 0.41	0.04
Pediatric Dentistry	5.50 ± 1.22	5.50 ± 0.84	0.92	2.67 ± 0.81	2.33 ± 0.81	0.33
Periodontology	5.50 ± 0.83	2.30 ± 0.70	0.001	2.50 ± 0.54	1.33 ± 0.51	0.01
Preventive Dentistry	4.80 ± 0.98	4.83 ± 1.02	0.50	2.17 ± 0.75	1.17 ± 0.40	0.02
Prosthodontics	4.30 ± 1.60	4.00 ± 2.44	0.86	2.17 ± 0.98	1.5 ± 0.54	0.20
Oral Surgery	6.00 ± 0.00	4.85± 1.94	0.14	2.67 ± 0.51	1.83 ± 0.40	0.02
Overall	5.29 ± 1.1/6.0	4.5 ± 1.7/5.0	0.024	2.44 ± 0.71/3.0	1.69 ± 0.62/2.0	<0.001

## Data Availability

The original contributions presented in this study are included in the article. Further inquiries can be directed to the corresponding author.

## Abbreviations

The following abbreviations are used in this manuscript:

AI	Artificial Intelligence
ChatGPT	Chatbot Generative Pre-Trained Transformer
GPT-3.5	Generative Pre-Trained Transformer, version 3.5
GPT-4	Generative Pre-Trained Transformer, version 4
SD	Standard Deviation
SPSS	Statistical Package for the Social Sciences
USMLE	United States Medical Licensing Examination

## Appendix A

The following tables in this appendix contain the questions and provided answers generated from ChatGPT 3.5 and 4.

**Table A1:** ChatGPT questions and answers about Endodontics.

Type	Difficulty	Question	ChatGPT 3.5	ChatGPT 4
Binary	Easy	Is rubber dam required in root canal treatment?	Rubber dam usage in root canal treatment is highly recommended and often considered a standard of care in dental practice. The American Association of Endodontists (AAE) and the American Dental Association (ADA) both advocate for the use of rubber dam isolation during root canal procedures. A rubber dam is a thin sheet of latex or non-latex material that is stretched over a frame and placed around the tooth being treated. It has small holes through which the individual tooth is exposed while isolating it from the rest of the oral cavity. The dam provides several benefits in root canal treatment: 1. Moisture control: Rubber dam creates a dry and isolated field, keeping the tooth clean and moisture-free during the procedure. It prevents contamination from saliva, blood, and oral bacteria, which is crucial for achieving optimal disinfection and sealing of the root canal system. 2. Infection control: By isolating the tooth, rubber dam significantly reduces the risk of bacteria from the oral cavity entering the root canal system. This helps in preventing potential post-treatment infections and improving the long-term success of the root canal therapy. 3. Patient safety: Rubber dam provides a barrier between the dental instruments, irrigants, and the patient’s oral tissues, minimizing the chances of accidental ingestion or aspiration of materials during the procedure. 4. Enhanced visibility and access: The use of rubber dam improves visibility by keeping the treatment area dry and isolated, allowing the dentist to clearly visualize the tooth and root canal anatomy. It also provides better access for instrumentation and placement of filling materials. While there may be specific situations where rubber dam placement is challenging or contraindicated, such as severe latex allergies or anatomical limitations, the benefits of its use generally outweigh any potential drawbacks. Dentists should consider patient comfort, safety, and infection control when determining whether to use rubber dam during root canal treatment, and they may use alternative isolation methods if appropriate. It’s important to consult with a dental professional for personalized advice regarding your specific dental treatment needs and considerations.	Rubber dam isolation is considered a standard of care in endodontics, including root canal treatment. The use of a rubber dam provides a number of benefits, including the prevention of aspiration or ingestion of instruments and materials, the protection of oral tissues from endodontic materials and instruments, and the creation of a clean and dry working environment. The American Association of Endodontists (AAE) strongly recommends the use of rubber dam isolation for all endodontic treatments. In the AAE’s “Clinical Considerations for a Regenerative Procedure,” it is stated that “Isolation with a rubber dam is mandatory to ensure that the canal system can be disinfected.” Similarly, the European Society of Endodontology (ESE) in their quality guidelines suggests that the “use of a rubber dam should be considered as mandatory in all cases of nonsurgical root canal treatment.” Furthermore, the American Dental Association (ADA) in its “Guidelines for the Use of Sedation and General Anesthesia by Dentists” states that for most dental procedures (including endodontic procedures), a dental dam should be used as a safety measure when any dental procedure is performed under sedation or general anesthesia. Therefore, while some practitioners may not use it in every case, the use of a rubber dam is widely accepted as a best practice and is recommended by major dental and endodontic organizations for root canal treatment. Always consult with a dental professional for personalized advice and care.
	Medium	Is copious chemical irrigation mandatory to achieve better outcomes in root canal treatment?	Copious chemical irrigation is considered a crucial step in root canal treatment to achieve better outcomes. Dental guidelines and recommendations emphasize the importance of using irrigants during root canal procedures. The American Association of Endodontists (AAE) and the European Society of Endodontology (ESE) provide guidance on the use of chemical irrigation in root canal treatment. Here are the reasons why copious chemical irrigation is often considered mandatory: 1. Antimicrobial action: Irrigants help eliminate bacteria, fungi, and other microorganisms present in the root canal system. The main irrigants used in root canal treatment are sodium hypochlorite (NaOCl) and chlorhexidine gluconate. Sodium hypochlorite is highly effective against bacteria and has the ability to dissolve organic tissue remnants. Chlorhexidine has broad-spectrum antimicrobial properties and can help in disinfecting the root canal system. 2. Tissue dissolution and debris removal: Irrigants aid in dissolving and flushing out organic tissue debris, pulp remnants, and dentinal shavings generated during instrumentation. This assists in thoroughly cleaning the root canal system and promoting optimal disinfection. 3. Smear layer removal: During root canal instrumentation, a layer called the smear layer is created on the walls of the root canal. The smear layer contains organic and inorganic debris that can harbor bacteria. Chemical irrigation helps in removing or reducing the smear layer, enhancing the penetration of intracanal medicaments and the obturation material. 4. Lubrication and canal negotiation: Irrigants act as lubricants, facilitating the smooth movement of endodontic instruments within the root canal. This aids in negotiating curved canals and reducing the risk of instrument fracture. While the use of copious chemical irrigation is generally recommended, it is important to exercise caution and follow appropriate protocols. Dentists should consider factors such as the concentration and volume of the irrigants, potential interactions with other materials, and the patient’s medical history to ensure safe and effective irrigation. It’s worth noting that individual cases may have specific considerations or contraindications for certain irrigants. Therefore, it’s essential to consult with a dental professional who can evaluate your specific situation and provide personalized recommendations for root canal treatment.	The use of chemical irrigation is a crucial step during root canal treatment. The main objectives of chemical irrigation in endodontics are to dissolve organic tissues, kill bacteria in the root canal system, remove the smear layer, and to help in shaping and cleaning the root canal. The American Association of Endodontists (AAE) in its Clinical Practice Guidelines emphasizes the importance of irrigation during root canal treatment. However, the AAE does not specifically mention “copious” irrigation, but it is commonly accepted that sufficient volume of irrigant should be used to facilitate cleaning and disinfection throughout the treatment. Commonly used irrigants include: 1. Sodium Hypochlorite (NaOCl): It’s the most commonly used irrigant due to its ability to dissolve organic tissue and its potent antimicrobial activity. The AAE recommends the use of NaOCl at concentrations ranging from 0.5% to 5.25%. 2. EthyleneDiamine TetraAcetic Acid (EDTA): Usually used to remove the smear layer (the layer of debris remaining on the canal walls after instrumentation), especially in the final stages of root canal preparation. 3. Chlorhexidine (CHX): It’s an alternative to NaOCl, especially for patients with an allergy to NaOCl. CHX has broad-spectrum antimicrobial activity, but it doesn’t dissolve organic tissues. The AAE guidelines also mention the use of ultrasonic activation or sonic activation to enhance the effectiveness of irrigation, a technique known as passive ultrasonic irrigation (PUI) or passive sonic irrigation (PSI). In conclusion, while the term “copious” is not specifically mentioned in the guidelines, the consensus is that sufficient irrigation is essential for better outcomes in root canal treatment. The volume and type of irrigant, along with the use of activation techniques, should be selected based on the individual case and the clinical judgement of the dentist.
	Hard	Does the root canal system need to be filled with medication between appointments?	The need to fill the root canal system with medication between appointments in root canal treatment depends on various factors, including the complexity of the case, presence of infection, and the treatment approach chosen by the dentist. Dental guidelines and recommendations provide guidance on the use of intracanal medicaments in root canal therapy. In some cases, it is common practice to place intracanal medication between appointments, particularly in cases with active infections or when a multi-visit treatment approach is employed. The goals of using intracanal medicaments are to disinfect the root canal system, alleviate symptoms, and promote healing. Here are some scenarios where intracanal medication may be used: 1. Active infection: If there is evidence of an active infection, such as a large periapical lesion or persistent symptoms, an intracanal medication may be placed between appointments to help control the infection. This medication can help eliminate bacteria and reduce inflammation within the root canal system. 2. Multi-visit treatment approach: In certain cases, a multi-visit treatment approach may be preferred. This approach involves leaving the root canal system medicated between visits to allow for sufficient disinfection and healing. Intracanal medicaments can help maintain a sterile environment within the root canal system during the healing phase. 3. Pulpal revascularization procedures: In cases where regenerative procedures, such as pulpal revascularization, are performed, intracanal medication may be used to promote the growth of new tissue and aid in the regeneration process. It’s important to note that not all cases require intracanal medication between appointments. In some situations, immediate obturation of the root canal system may be appropriate, particularly when the infection is adequately controlled and the canal is thoroughly cleaned and shaped. The decision to use intracanal medicaments and the choice of specific medicaments should be based on the individual patient’s needs and the professional judgment of the treating dentist. To ensure the best outcome, it is crucial to follow the guidance of a dental professional who can evaluate your specific case, taking into account factors such as the severity of infection, treatment goals, and overall oral health.	Intracanal medicaments, also known as interappointment dressings, can be used between appointments in root canal treatment for several reasons, such as to reduce the number of bacteria in the root canal, to prevent overgrowth of bacteria between appointments, and to alleviate symptoms. However, whether or not to use an interappointment dressing, and which medicament to use, depends on the specifics of the case. The American Association of Endodontists (AAE) in their Clinical Practice Guidelines, while not mandating the use of intracanal medicaments in all cases, do highlight their potential benefits. The guidelines note that intracanal medicaments can be beneficial in managing symptoms and reducing microbial load, particularly in cases of apical periodontitis. Commonly used intracanal medicaments include: 1. Calcium Hydroxide: It is widely used due to its antimicrobial properties and ability to inactivate endotoxins. It is especially beneficial in cases of necrotic pulp and apical periodontitis. 2. Chlorhexidine Gel: It has a broad-spectrum antimicrobial activity and is often used in cases of resistant microbial infection. 3. Triple Antibiotic Paste: A mixture of metronidazole, ciprofloxacin, and minocycline. It’s often used in cases of severe infection or in regenerative endodontic procedures. While the use of intracanal medicaments can be beneficial in certain cases, it’s not always necessary, especially in cases where complete cleaning and shaping can be achieved in one visit and there are no signs of infection. The decision should be made based on the individual case and the clinical judgement of the dentist. Always consult with a dental professional for personalized advice and care.
Descriptive	Easy	What are the indications for root end surgery?	Root end surgery, also known as apicoectomy, may be indicated in cases where traditional root canal treatment has failed or when retreatment is not possible. This may include: 1. Persistent symptoms or infection after root canal treatment. 2. Presence of a cyst or granuloma at the root tip. 3. Blocked or curved root canals that cannot be adequately treated. 4. Fractured or damaged root tips that cannot be repaired. 5. Tooth root that is too short for a crown or other restoration. 6. Access to the root canal is obstructed by a post or other obstruction. It is important to discuss all treatment options with your endodontist and follow any recommended guidelines for successful root end surgery.	Root end surgery, also known as an apicoectomy or endodontic microsurgery, is a surgical procedure that is performed to treat issues in the roots of teeth. An apicoectomy is typically considered when a conventional root canal therapy has failed, or when a re-treatment of a root canal is not advisable or possible. The following are some specific indications for root end surgery: 1. Persistent Infection or Disease: If a tooth still has signs of infection or disease following root canal treatment, root end surgery may be necessary. This could be due to complex anatomy of the root canals that was not entirely cleaned during the initial procedure. 2. Failed Root Canal Treatment: This can occur due to a variety of issues, including undetected canal branches, complex root anatomy, or a persistent infection that is not resolved with conventional root canal therapy. 3. Non-Healing Endodontic Lesion: When a radiolucent lesion associated with a root-filled tooth does not heal or decrease in size after conventional root canal treatment, a root end surgery may be indicated. 4. Root Resorption: This is a process where the tooth’s structure is broken down and absorbed by the body. It can occur due to a variety of causes and may necessitate root end surgery if it affects the tooth’s root. 5. Tooth Root Damage or Fracture: If a tooth’s root is damaged or fractured, root end surgery might be required to remove the affected portion and restore health to the surrounding tissues. 6. Retreatment Not Possible or Advisable: If retreatment of a root canal is not possible due to a variety of reasons (like inaccessible canals, significant restorations, or posts), or if retreatment might risk further damage to the tooth or surrounding structures, root end surgery might be the preferred option. 7. Extrusion of Filling Material: If the filling material from a root canal treatment has been extruded into the periapical tissues, an apicoectomy may be necessary to remove the extruded material. 8. Inability to Obtain a Seal Due to Root Canal Obstruction: If the root canal is obstructed by a broken instrument, calcification, or any other obstruction, and this prevents the ability to obtain a proper seal during root canal therapy, then apicoectomy may be indicated. It’s important to note that the decision to perform root end surgery should be made in consultation with the patient, and after considering other treatment options, like retreatment or extraction and replacement of the tooth. The patient’s overall health, the prognosis of the tooth, the cost, and the patient’s preferences should all be considered in the decision-making process.
	Medium	How can the technical quality of root canal treatment be determined?	The technical quality of root canal treatment can be determined through various methods, including clinical examination, radiographic evaluation, and use of specialized tools or techniques such as cone beam computed tomography (CBCT) or electronic apex locator. The American Association of Endodontists has established guidelines and standards for evaluating the technical quality of root canal treatment, which may include criteria such as the presence of sufficient filling material, proper length and taper of the canal, and absence of any voids or gaps. Your endodontist will assess these factors to determine the overall quality of your root canal treatment.	The technical quality of root canal treatment (RCT) can be assessed by considering several important factors. These factors are generally evaluated through clinical examination and radiographic analysis. According to the guidelines set by the American Association of Endodontists (AAE) and the European Society of Endodontology (ESE), the following aspects should be evaluated: 1. Cleaning and Shaping of the Root Canal System: The root canal system should be thoroughly cleaned and appropriately shaped to allow for sufficient disinfection and filling. The canal should follow the natural curvature of the root. Over-preparation or under-preparation of the canal can lead to a poor prognosis. 2. Length of the Root Canal Filling: The root canal filling should extend to the radiographic apex of the tooth. Ideally, it should stop within 0–2 mm short of the radiographic apex. Overfilling can potentially cause damage to the periapical tissues, while underfilling may leave spaces for bacteria to multiply. 3. Density of the Root Canal Filling: The filling should be compact and without voids, which can be a potential space for bacterial growth. The canal should be uniformly filled from the coronal to the apical end. 4. Absence of Iatrogenic Errors: There should be no procedural errors such as ledging, transportation, perforation, or instrument separation. 5. Coronal Seal: The coronal seal should prevent the re-entry of bacteria from the oral cavity into the periapical area. An inadequate coronal seal is a common cause of endodontic failure. 6. Radiographic Healing: Follow-up radiographs are an essential part of assessing the quality of RCT. Healing is indicated by a reduction in the size of any pre-existing radiolucent area associated with the root of the treated tooth. 7. Clinical Symptoms: Absence of clinical symptoms such as pain, swelling, sinus tract, or mobility of the tooth is also a good indication of successful treatment. 8. Periodontal Health: The tooth should not show signs of increased pocket depth or bleeding on probing post-treatment. 9. Functional Outcomes: The tooth should be functional without pain or discomfort during mastication. It’s important to remember that achieving these technical aspects of a good-quality root canal treatment is only one part of successful endodontic therapy. The body’s ability to heal and the control of other factors like the patient’s overall health, oral hygiene, and the control of other oral diseases are just as critical for the long-term success of the treatment.
	Hard	What is the proper technique used to remove posts within root canal system?	The proper technique used to remove posts within the root canal system may vary depending on the type and location of the post. In general, it involves: 1. Accessing the post by creating a small opening in the tooth or by removing a portion of the crown. 2. Loosening the post by using ultrasonic instruments or heat. 3. Grasping and removing the post with specialized instruments, such as pliers or trephines. 4. Cleaning and shaping the remaining root canal space to ensure proper filling and sealing. 5. Evaluating the remaining tooth structure and determining the best restoration option. It is important to follow proper techniques and guidelines to avoid damaging the tooth or surrounding structures during post removal.	Removing a post from the root canal system can be a complex procedure requiring careful planning and execution to avoid damaging the tooth structure or the surrounding tissues. Here is a general outline of the technique, incorporating guidelines from the American Association of Endodontists (AAE): 1. Assessment: Evaluate the type, size, length, and location of the post, as well as the remaining tooth structure, through radiographs and clinical examination. This will help in planning the removal strategy. 2. Access Preparation: If the post is covered by a crown, carefully remove the crown to expose the post. This may sometimes involve sectioning the crown. If the crown cannot be removed, an access hole may need to be created. 3. Post Loosening: Depending on the type of post (cast post and core, prefabricated metal post, or fiber post), different techniques may be used to loosen it: - For metal posts, ultrasonic devices can be used to vibrate the post and break down the luting cement. They can also be used to create a groove in the post to enhance the grip of post removal instruments. - For fiber posts, they can often be removed by creating a groove with a diamond bur and using post removal instruments. 4. Post Removal: Once the post has been loosened, use a post removal instrument or a dental extraction forceps to gently remove the post. In some cases, a specialized kit like the Masserann kit or the Ruddle Post Removal System can be used. 5. Root Canal Treatment: After post removal, the remaining root canal filling material needs to be removed, and the root canal system should be properly cleaned, shaped, and disinfected before refilling. 6. Restoration: After root canal treatment, assess the remaining tooth structure. If sufficient tooth structure remains, the tooth can be directly restored with a filling material and a crown. If the remaining tooth structure is inadequate, a new post and core may need to be placed, followed by a crown. Please note that this is a technically demanding procedure that presents risks, such as root fracture or perforation. Therefore, it should only be performed by a skilled and experienced practitioner. The patient should be informed about all potential risks and benefits, and alternative treatment options, like extraction and replacement of the tooth, should be considered.

**Table A2:** ChatGPT questions and answers about Periodontics.

Type	Difficulty	Question	ChatGPT 3.5	ChatGPT 4
Binary	Easy	Would a gingvectomy be suitable instead of crown lengthening in cases with no periodontal pockets and no bone loss?	In cases with no periodontal pockets and no bone loss, a gingivectomy may be a suitable alternative to crown lengthening. A gingivectomy is a surgical procedure that involves removing excess gum tissue, which can help to expose more of the tooth structure and improve the appearance of the gums. However, it is important to consult with your dentist or periodontist to determine the most appropriate treatment option for your specific case. In some cases, a combination of both procedures may be necessary for optimal results.	“Crown lengthening and gingivectomy are both procedures used in dentistry to expose more of the tooth’s surface. However, they are used for different indications and have different implications. A gingvectomy is a procedure that involves the removal of gingival tissue and is typically indicated for the reduction of enlarged gingiva (gingival hyperplasia) or to eliminate periodontal pockets in cases of periodontal disease. Crown lengthening, on the other hand, is a surgical procedure wherein both the gingival tissue and the underlying bone are recontoured to expose more of the tooth structure. This procedure is often required when a tooth needs a new crown or other restoration and there isn’t enough tooth structure above the gum line to support it (due to deep decay, tooth fracture, etc.), or to improve the aesthetics of a “gummy smile”. The American Academy of Periodontology (AAP) guidelines suggest that in the absence of periodontal pockets and bone loss, a gingivectomy can be an appropriate treatment to expose more of the tooth structure if only soft tissue removal is required. In such cases, a gingivectomy can expose sufficient tooth structure for restorative procedures while maintaining an adequate biological width (the space between the base of the gingival sulcus and the alveolar bone), ensuring periodontal health. It is a less invasive procedure compared to crown lengthening and usually has a shorter healing time. However, if the amount of tissue that needs to be removed would encroach upon the biological width (usually at least 2 mm is required to maintain periodontal health), a crown lengthening procedure would be necessary to avoid future periodontal problems. In crown lengthening, bone is also removed to maintain this necessary space. The decision between gingivectomy and crown lengthening should be made after a thorough clinical and radiographic evaluation, considering the patient’s overall oral health, the specific tooth’s condition, the amount of tooth structure that needs to be exposed, and the need to maintain biological width. It’s also essential to discuss the procedure, its benefits, risks, and alternatives with the patient.”
	Medium	Is there certain type of bonydefects that are most favourable for GTR results?	“Yes, certain types of bony defects may be more favorable for guided tissue regeneration (GTR) results. These may include:	“Guided tissue regeneration (GTR) is a surgical dental procedure used to stimulate the growth of new alveolar bone, cementum, and periodontal ligament around teeth affected by periodontal diseases. The American Academy of Periodontology (AAP) and other professional dental organizations have provided guidelines and recommendations on when GTR may be most effective. The type of bony defect does indeed play a significant role in the success of GTR. Here are the types of defects that have been found to respond most favorably: 1. Intrabony (vertical) Defects: These defects consist of a vertical osseous component and have been shown to respond well to GTR. In particular, deep (4–6 mm), narrow intrabony defects with at least one intact bony wall are found to be most favorable. 2. Furcation Involvements: GTR can be used for treating furcation involvements, especially for Class II furcations, where there is a horizontal loss of periodontal tissue support interradicularly with the furcation area remaining coronal to the alveolar bone. 3. Localized Defects: GTR has proven to be more successful in localized defects rather than generalized aggressive periodontitis. It should be noted that while the type of bony defect is a crucial factor in the success of GTR, other factors also contribute to the prognosis. These include the patient’s overall health, oral hygiene, smoking status, and the ability to maintain regular periodontal maintenance care. Furthermore, GTR is a technically sensitive procedure, and the skill and experience of the practitioner also significantly impact the outcome. As always, the decision to perform GTR should be made after a thorough evaluation of the patient and the presentation of all treatment options, along with their benefits, risks, and implications.”
	Hard	Is local antibiotics preferred over systemic antibiotics in treating cases with genarlised periodontitis?	Local antibiotics may be preferred over systemic antibiotics in treating cases with generalized periodontitis. This is because local antibiotics can be directly applied to the affected area, providing a higher concentration of medication and reducing the risk of side effects. However, the use of antibiotics in periodontal treatment should be carefully evaluated and prescribed in accordance with the American Academy of Periodontology’s guidelines to ensure proper management and minimize the risk of antibiotic resistance. Your periodontist will assess your individual case and determine the most appropriate treatment plan for you.	“The American Academy of Periodontology (AAP) and the European Federation of Periodontology (EFP) have provided guidelines on the use of antibiotics in the management of periodontal diseases. The decision to use local or systemic antibiotics should be made based on the severity and extent of the disease, the patient’s health status, and the response to mechanical periodontal therapy. 1. Systemic Antibiotics: Systemic antibiotics are typically reserved for aggressive forms of periodontitis, or for patients with generalized severe periodontitis that has not responded adequately to non-surgical periodontal therapy (scaling and root planing). They can also be considered in patients with systemic conditions that may affect periodontal healing, such as diabetes or immunosuppressive conditions. 2. Local Antibiotics: Local antibiotics (also known as local drug delivery) can be an adjunctive treatment to scaling and root planing in managing localized periodontal pockets or isolated areas of periodontitis. They are typically not used as a primary treatment for generalized periodontitis. It’s important to note that antibiotics (both systemic and local) are adjunctive therapy and should not replace mechanical periodontal therapy, which is the first line of treatment for periodontitis. Moreover, inappropriate or overuse of antibiotics can lead to antibiotic resistance, which is a significant public health concern. Therefore, the decision to use antibiotics should be made carefully, considering the potential benefits and risks. In summary, for generalized periodontitis, the primary treatment is comprehensive periodontal therapy, including scaling and root planing, possibly combined with systemic antibiotics if the disease is severe or not responsive to mechanical therapy alone. Local antibiotics are more typically used for treating localized or isolated periodontal defects. As always, individual patient factors and response to treatment should guide therapy.”
Descriptive	Easy	What is occlusal truma? What are the common clinical features to occlusal trauma?	“Occlusal trauma is a condition where excessive or abnormal forces are applied to the teeth and supporting structures, leading to damage or injury. This can be caused by factors such as malocclusion, bruxism (teeth grinding), or improper use of dental restorations. Some common clinical features of occlusal trauma may include: 1. Tooth mobility or looseness. 2. Tooth wear, chipping, or fractures. 3. Sensitivity to hot, cold, or pressure. 4. Gum recession or pocket formation. 5. Pain or discomfort in the jaw or facial muscles. 6. Changes in the bite or misalignment of teeth. 7. Enlargement or discomfort of the temporomandibular joint (TMJ). 8. Difficulty chewing or speaking. 9. Headaches or earaches. 10. Limited mouth opening or jaw locking. It is important to diagnose and treat occlusal trauma to prevent further damage and maintain oral health. Your dentist may recommend various treatment options, such as occlusal adjustments, splints, or orthodontic treatment, depending on the severity and cause of the trauma.”	“Occlusal trauma refers to tissue injury resulting from excessive force or pressure on the teeth and surrounding structures due to malocclusion, bruxism (teeth grinding), or other conditions leading to abnormal bite forces. It is classified into two types: 1. Primary Occlusal Trauma: This occurs when excessive occlusal force is applied to a tooth with a healthy periodontium. This could be due to habits like bruxism or the presence of high points in restorations or prostheses. 2. Secondary Occlusal Trauma: This occurs when normal or excessive occlusal forces result in injury to a tooth with a compromised periodontium, typically due to periodontitis where there is bone loss and increased tooth mobility. The American Academy of Periodontology (AAP) and other dental associations have outlined common clinical features of occlusal trauma. They include: 1. Tooth Mobility: The affected tooth may show increased mobility due to the pressure exerted on it. The mobility may be more apparent in the case of secondary occlusal trauma. 2. Pain on Occlusion: The patient may experience pain or discomfort when biting or chewing. 3. Tooth Migration: Changes in the position of the teeth can occur over time due to the excessive force. 4. Enlargement of the Periodontal Ligament (PDL) Space: Radiographically, there might be widening of the periodontal ligament space. 5. Root Resorption: In some chronic cases, the body’s attempt to remodel the tooth structure to accommodate the forces can lead to root resorption. 6. Alveolar Bone Changes: Radiographic examination may show increased density of the alveolar bone (sclerosis) or irregular bone loss. 7. Tooth Wear: Excessive wear facets may be visible on the occlusal surfaces of the teeth, particularly in cases of bruxism. 8. Fractures: Excessive forces can lead to tooth fractures or cracks. It’s important to note that while occlusal trauma can cause these signs and symptoms, they are not exclusive to occlusal trauma and can be seen in other dental conditions as well. A thorough diagnosis should be made considering all clinical and radiographic findings. Treatment should aim at eliminating the cause of trauma, which could involve occlusal adjustment, splinting of mobile teeth, use of a nightguard in cases of bruxism, or orthodontic treatment in cases of malocclusion.”
	Medium	What are the indications for surgical root covarage and what techniques could be used ?	“Surgical root coverage, also known as gingival grafting, is performed to correct gum recession and restore gum tissue around the tooth. It may be indicated in cases where there is significant gingival recession, which can lead to tooth sensitivity, root exposure, and esthetic concerns. Some techniques that may be used for surgical root coverage include: 1. Connective tissue grafting: This involves taking a small piece of tissue from the roof of the mouth and attaching it to the area of recession. 2. Free gingival grafting: Similar to connective tissue grafting, but the tissue is taken directly from the gum tissue around the tooth. 3. Pedicle grafting: This technique uses existing gum tissue near the recession area and rotates it to cover the exposed root. 4. Alloderm grafting: This involves using a donor tissue graft instead of using the patient’s own tissue. Your periodontist will determine the most suitable technique for your specific case based on various factors such as the extent of recession, oral health, and esthetic goals.”	“Surgical root coverage procedures are indicated to treat gingival recession, which is the exposure in the roots of the teeth caused by a loss of gum tissue and/or retraction of the gingival margin from the crown of the teeth. The American Academy of Periodontology (AAP) suggests the following indications for surgical root coverage: 1. Esthetic Concerns: Patients may be dissatisfied with the appearance of their smile due to exposed root surfaces. 2. Root Sensitivity: Exposed root surfaces may lead to heightened sensitivity to hot, cold, or sweet substances. 3. Root Caries or Abrasion Lesions: Exposed root surfaces are more susceptible to decay and abrasive wear. 4. Inadequate Attached Gingiva: To increase the width of keratinized tissue or attached gingiva in areas of recession. 5. Prevent Further Recession: In cases where progressive recession has been noted. The following techniques can be used for root coverage, depending on the specific circumstances and needs of the patient: 1. Coronally Advanced Flap (CAF): This is a common technique where a flap of gingival tissue is mobilized and moved coronally (towards the crown of the tooth) to cover the exposed root surface. 2. Subepithelial Connective Tissue Graft (SCTG): This technique involves the harvesting of connective tissue from another site in the mouth (usually the palate), which is then placed under a coronally advanced flap at the site of recession. 3. Free Gingival Graft (FGG): This involves the transfer of a thin piece of tissue from the palate to the area of recession. 4. Acellular Dermal Matrix Allograft (ADMA): This is an alternative to SCTG that utilises donated human dermal tissue, which can avoid the need for a second surgical site. 5. Guided Tissue Regeneration (GTR): This technique involves placing a resorbable or non-resorbable barrier to allow for growth of the periodontal ligament and bone while preventing the faster-growing epithelium from filling in the defect. 6. Xenogeneic Collagen Matrix (XCM): This technique uses a collagen matrix derived from a different species to serve as a scaffold for soft tissue regeneration. The choice of technique depends on various factors, including the number and location of the teeth involved, the amount of keratinized tissue present, patient preference, and clinician expertise. Also, it’s important to manage factors contributing to the recession, such as traumatic tooth brushing habits or occlusal trauma, to ensure the long-term success of the treatment. As always, patients should be informed about the procedure, its benefits, risks, and alternatives.”
	Hard	How you assess and grade teeth with furcation involvment ?	“The assessment and grading of teeth with furcation involvement can be done using various methods, including: 1. Probing depth: A periodontal probe is used to measure the depth of the pocket between the tooth and gum tissue at the furcation area. 2. Radiographic evaluation: X-rays can help to assess the bone and root morphology in the furcation area. 3. Clinical examination: The clinician visually inspects the furcation area for any signs of inflammation, recession, or exposure of the root surface. 4. Classification systems: Various classification systems, such as the Hamp classification or the Furcation Involvement and Its Treatment (FIST) index, can be used to grade the severity of furcation involvement based on factors such as the depth and extent of the lesion, root morphology, and bone loss. Your dentist or periodontist will use these methods to assess and grade the severity of furcation involvement, which will help to determine the most appropriate treatment approach for your specific case.”	“Furcation involvement refers to the destruction of the alveolar bone between the roots of a multi-rooted tooth (such as molars and premolars), as a result of periodontal disease. This can make oral hygiene difficult, and it increases the risk of further periodontal breakdown. The American Academy of Periodontology (AAP) utilizes a classification system to grade the severity of furcation involvement: 1. Grade I Furcation Involvement: Early or incipient involvement where there is bone loss within the furcation area but the furcation cannot be probed clinically. The furcation is not clinically visible but can be detected using a Nabers probe or by radiographic examination. 2. Grade II Furcation Involvement: Moderate involvement in which some portion of the furcation is still covered by alveolar bone and the probe cannot pass completely through the furcation. It’s characterized by a definite horizontal bone loss, and the furcation can be clinically probed, but not all the way through. 3. Grade III Furcation Involvement: Severe involvement where the furcation is fully exposed and the probe can pass completely through the furcation. It’s characterized by horizontal through-and-through destruction of the alveolar bone. 4. Grade IV Furcation Involvement: Similar to Grade III, but with the additional feature of recession of the gingival margin, making the furcation opening clinically visible. It’s worth noting that furcation involvement is usually more severe on the radiograph than what is clinically apparent due to the presence of gingival tissue covering the furcation area. Furthermore, while the classification is useful in assessing the extent of the furcation involvement, it does not necessarily dictate the prognosis of the tooth. The prognosis of a tooth with furcation involvement is also affected by other factors, such as the patient’s oral hygiene, the depth of the periodontal pockets, the extent of attachment and bone loss, and the patient’s overall health. Treatment options can range from non-surgical periodontal therapy, local drug delivery in the furcation area, regenerative procedures, root resection, or even extraction in severe cases. The choice of treatment should be based on a comprehensive evaluation of the patient’s condition and discussed thoroughly with the patient.”

**Table A3:** ChatGPT questions and answers about Preventive Dentistry.

Type	Difficulty	Question	ChatGPT 3.5	ChatGPT 4
Binary	Easy	Are general socio-economic, cultual and enviromental conditions among determenants of oral health?	Yes, general socio-economic, cultural, and environmental conditions can be significant determinants of oral health. These factors can affect access to dental care, oral hygiene practices, and exposure to risk factors for oral diseases. For example, individuals from lower socio-economic backgrounds may have limited access to dental services and may also face challenges in maintaining good oral hygiene due to financial constraints or lack of education. Cultural practices and beliefs may also impact oral health, such as diets high in sugar or tobacco use. Environmental factors, such as exposure to fluoride in water or air pollution, can also have an impact on oral health. It is essential to consider these determinants when promoting oral health and developing public health policies and programs.	“Yes, socio-economic, cultural, and environmental conditions are indeed significant determinants of oral health. The World Health Organization (WHO) and various dental associations like the American Dental Association (ADA) and the American Association of Public Health Dentistry (AAPHD) acknowledge this in their guidelines and policies. 1. Socio-economic Conditions: Socio-economic status, including income level and education, significantly impacts oral health. Lower socio-economic status has been linked to higher rates of dental decay, periodontal disease, and tooth loss. These populations often have limited access to dental care due to financial constraints, lack of insurance, and limited availability of dental providers in low-income areas. 2. Cultural Factors: Cultural beliefs and practices play a key role in oral health behaviors, including dietary habits, oral hygiene practices, attitudes towards dental care, and use of traditional or alternative therapies. For example, certain cultural practices may contribute to increased sugar consumption, leading to higher rates of dental caries. Understanding cultural factors is crucial in providing culturally competent dental care and in developing effective oral health promotion strategies. 3. Environmental Conditions: Environmental factors, such as fluoridation of community water supplies, can significantly impact oral health. Access to fluoride, either through water, toothpaste, or professional applications, is a key factor in preventing dental caries. Other environmental factors include the availability and accessibility of healthy food options, tobacco and alcohol advertising, and the physical environment, which can influence physical activity levels and, consequently, overall health status. These determinants can interact and cumulatively impact a person’s oral health over their lifespan. Thus, addressing oral health inequalities requires a broad public health approach that considers all these determinants. It’s not enough to just focus on individual behaviors; policies and interventions should aim at improving social, economic, cultural, and environmental conditions to promote oral health for all.”
	Medium	Is tooth paste more effective in reducing DMFT compared to fluoride varnish?	Both toothpaste and fluoride varnish are effective in reducing dental caries and improving oral health. However, the effectiveness may vary depending on individual factors such as oral hygiene habits, diet, and fluoride exposure. According to the American Dental Association, toothpaste with fluoride is considered the most effective form of fluoride for preventing tooth decay. Fluoride varnish, on the other hand, is a concentrated form of fluoride that is applied directly to the teeth by a dental professional. It can provide a higher concentration of fluoride and may be more suitable for individuals at high risk of tooth decay. Your dentist will determine the most appropriate form of fluoride for your specific needs.	“Dental caries is a multifactorial disease affected by various factors, including oral hygiene habits, diet, and fluoride exposure. The DMFT index (Decay, Missing due to decay, Filled Teeth) is a common measure used in dental research to assess the prevalence and severity of dental caries. Fluoride toothpaste and fluoride varnish are both effective methods for delivering fluoride to the teeth to prevent and control dental caries. They work by enhancing remineralization of early carious lesions and inhibiting demineralization of tooth enamel. The American Dental Association (ADA) and the Centers for Disease Control and Prevention (CDC) recommend the use of both fluoride toothpaste and fluoride varnish as part of a comprehensive approach to caries prevention. Therefore, it’s not so much a question of one being more effective than the other, but rather how they can be used together for maximum benefit. 1. Fluoride Toothpaste: Daily tooth brushing with fluoride toothpaste is considered the cornerstone of caries prevention. It provides regular, low-dose exposure to fluoride. The ADA recommends brushing twice a day with a fluoride toothpaste. 2. Fluoride Varnish: This is a high-concentration fluoride product that is professionally applied to the tooth surfaces. It has been shown to be effective in reducing caries in both primary and permanent teeth. The ADA and the CDC recommend the application of fluoride varnish in children every 3–6 months, depending on caries risk. It’s important to note that the effectiveness of these interventions can be influenced by factors such as patient compliance (i.e., regular brushing with fluoride toothpaste) and access to professional dental care for fluoride varnish applications. Also, these interventions should be supplemented with other preventive measures such as a balanced diet low in sugar, regular dental check-ups, and dental sealants in the case of children and adolescents. In conclusion, both fluoride toothpaste and fluoride varnish play important roles in caries prevention, and their combined use can provide the most effective reduction in DMFT scores. As always, individual recommendations should be based on the patient’s age, caries risk, and other individual factors.”
	Hard	Is fissure sealent recommended as public health mesure?	Yes, fissure sealants are recommended as a public health measure to prevent dental caries, especially in children. Fissure sealants are thin, plastic coatings that are applied to the deep grooves and pits on the chewing surfaces of teeth, creating a barrier that protects against bacteria and food particles. They are most commonly used on permanent molars, which are at a higher risk of developing cavities due to their anatomy. The Centers for Disease Control and Prevention (CDC) lists fissure sealants as one of the most effective ways to prevent tooth decay and recommends their use in school-based and community-based programs to promote oral health.	“Yes, dental sealants are widely recommended as an effective public health measure for the prevention of dental caries in children and adolescents. They are particularly effective in preventing caries in the pits and fissures of molars, which are areas prone to decay and difficult to clean with brushing. The American Dental Association (ADA), the American Academy of Pediatric Dentistry (AAPD), and the Centers for Disease Control and Prevention (CDC) endorse the use of dental sealants: 1. ADA: Recommends that sealants be placed on primary and permanent molars in children and adolescents soon after the tooth erupts and as long as the tooth remains caries-free. 2. AAPD: Recommends the application of pit and fissure sealants on the occlusal surfaces of primary molars in high-risk children as soon as the tooth’s anatomy allows complete sealant placement. 3. CDC: Recommends school-based sealant programs for children, particularly those from low-income families who are less likely to receive private dental care. The application of sealants is a simple procedure that can be performed by a dentist, dental hygienist, or other qualified dental professional, and it does not require drilling or removing tooth structure, making it a safe and painless process. However, in implementing a public health measure like a sealant program, several factors need to be considered: - Access to Care: Sealant programs should target populations with limited access to dental care, such as low-income or rural communities. - Training and Resources: Dental professionals need to be properly trained on the application of sealants, and the necessary resources should be available. - Follow-up and Maintenance: Regular check-ups are necessary to ensure that the sealants remain intact and effective. In conclusion, dental sealants are an effective and recommended public health measure for the prevention of dental caries in children and adolescents. They should be incorporated into comprehensive preventive strategies that also include fluoride exposure (through water, toothpaste, or professional applications), dietary counseling, and promotion of good oral hygiene practices.”
Descriptive	Easy	What is the optimal level of fluoride in water for anticariogenticity?	The optimal level of fluoride in water for anticariogenicity, or the prevention of dental caries, is 0.7 milligrams per liter (mg/L) according to the U.S. Public Health Service recommendation. This level is based on the premise that fluoride has a dose-dependent effect, meaning that a certain amount of fluoride is necessary for caries prevention, but too much fluoride can lead to fluorosis (a cosmetic condition that affects the appearance of teeth). The CDC also recommends monitoring the fluoride levels in drinking water to ensure it remains within the optimal range for optimal oral health benefits.	“The optimal level of fluoride in community water supplies for the prevention of dental caries has been a topic of extensive research and discussion. Fluoride in drinking water can provide significant benefits in reducing the prevalence and severity of dental caries. According to the U.S. Public Health Service (USPHS), as of their most recent recommendation in 2015, the optimal fluoride concentration in drinking water for caries prevention is 0.7 milligrams per liter (mg/L), or parts per million (ppm). This recommendation is a change from the previously recommended range of 0.7 to 1.2 mg/L. The change was made in response to an increase in sources of fluoride (such as toothpaste and other dental products) and the recognition of the risk of dental fluorosis in children from excessive fluoride exposure. The World Health Organization (WHO) also suggests a similar level. In areas with moderate climates, the WHO recommends a fluoride concentration of 1.0 mg/L, but this can be adjusted based on the average maximum daily air temperature. In hotter climates where water consumption may be higher, the optimal fluoride concentration may be lower to prevent excessive fluoride intake. It’s important to note that while fluoridation of community water supplies is a cost-effective public health measure to reduce dental caries, it should be complemented by other preventive strategies such as use of fluoride toothpaste, healthy diet, and regular dental check-ups. Also, the implementation of water fluoridation requires careful monitoring to ensure the optimal level of fluoride and to prevent overexposure. Lastly, it’s crucial to consider the local context, including dietary habits, climate, access to other sources of fluoride, and prevalence of dental caries, when determining the optimal level of fluoride in community water supplies.”
	Medium	what are the differences between a whole poulation approach, high risk and targeted community approach in the prevention of caries?	“A whole population approach, high-risk approach, and targeted community approach are three different strategies for preventing dental caries. A whole population approach involves implementing preventive measures, such as fluoridation of water and community-based programs, to benefit the entire population. This approach aims to reduce the overall burden of dental caries in the community. A high-risk approach focuses on identifying and targeting individuals who are at a higher risk of developing dental caries, such as children from low-income families or those with poor oral hygiene habits. This approach involves providing targeted interventions, such as fluoride varnish or sealants, to prevent caries in these high-risk individuals. A targeted community approach is a combination of both the whole population and high-risk approaches. It involves implementing preventive measures at a community level while also targeting high-risk individuals within that community. This approach aims to reduce the overall burden of dental caries in the community while also addressing the specific needs of high-risk individuals. The most effective approach may vary depending on the specific needs and resources of a community.”	“When it comes to caries prevention and control, different approaches can be implemented. These include the whole population approach, the high-risk strategy, and the targeted community approach. Each has its unique strengths and is suitable for different scenarios. 1. Whole Population Approach: This is also known as the universal strategy. In this approach, interventions are applied to the entire population without regard to individual risk status. An example is the fluoridation of community water supplies, a measure that benefits all individuals consuming the water. This approach can result in significant overall reduction in disease prevalence and is cost-effective at the population level. The main challenge is that it might overlook individual risk factors and may result in the delivery of interventions to individuals who might not need them. 2. High-Risk Strategy: This approach focuses on individuals or groups who are at a higher risk for dental caries based on identifiable risk factors. Interventions can be more intensive and tailored, such as increased frequency of fluoride varnish application or individual dietary counseling. This can result in a significant reduction in disease burden among those at highest risk. However, it requires proper risk assessment tools and can be resource-intensive. Additionally, as the majority of new cases may occur in individuals at moderate risk, this strategy alone may not result in a significant reduction in overall disease prevalence. 3. Targeted Community Approach: This strategy targets interventions to specific communities or groups that share common risk factors or characteristics, such as low-income communities or schools in disadvantaged areas. This approach allows for the efficient use of resources and can address health inequities by targeting groups with a higher burden of disease. An example would be school-based sealant programs in schools serving low-income communities. This approach requires good knowledge of community characteristics and needs and may not address inter-individual differences within the community. In practice, these strategies are not mutually exclusive and can be used in combination for a comprehensive approach to caries prevention. The choice of strategy should depend on factors such as the characteristics of the population, availability of resources, and the specific objectives of the prevention program. The American Dental Association (ADA), the American Academy of Pediatric Dentistry (AAPD), and the Centers for Disease Control and Prevention (CDC) all emphasize the importance of a comprehensive approach to caries prevention that includes elements of these strategies, tailored to the needs of the individual and the community.”
	Hard	Since caries level in the high-income countries are at their lowest levels ever recorded, do we still need water fluoridation and other community-based prevention programmes?	Yes, even though caries levels in high-income countries are at their lowest levels, water fluoridation and other community-based prevention programs are still necessary. This is because dental caries is a preventable disease, and without continued efforts, there is a risk of a resurgence in caries levels. Additionally, not all communities have access to preventive measures such as regular dental care or fluoridated water, so these programs help to address health disparities and promote oral health for all. It is important to continue with these prevention programs to maintain the progress made in reducing caries levels and to ensure optimal oral health for all individuals.	“While it is true that caries levels in high-income countries have decreased significantly over the decades, largely due to public health measures such as water fluoridation and the widespread use of fluoride toothpaste, it is important to note that dental caries remains a significant public health problem. The World Health Organization (WHO), the American Dental Association (ADA), and the Centers for Disease Control and Prevention (CDC), among others, continue to endorse water fluoridation and other community-based prevention programs for the following reasons: 1. Prevalence of Dental Caries: Even in high-income countries, dental caries is still prevalent, particularly among disadvantaged and vulnerable populations. Disparities in oral health persist, and caries can still impose a significant burden in terms of pain, suffering, and cost. 2. Cost-Effective Prevention: Water fluoridation is a cost-effective measure for caries prevention. It benefits all residents in a community regardless of age, socio-economic status, or access to dental care. Discontinuing it could lead to an increase in the prevalence and severity of dental caries. 3. Caries is a Multifactorial Disease: Caries is influenced by a variety of factors, including diet, oral hygiene habits, access to dental care, and socio-economic factors. Public health measures such as water fluoridation and school-based sealant programs help to address these factors at a population level. 4. Maintenance of Oral Health Gains: The gains in oral health that have been achieved through measures like water fluoridation and the use of fluoride toothpaste need to be maintained. Stopping these measures could lead to a rebound in caries levels. In conclusion, while we have made significant progress in reducing the level of dental caries, there is still a need for public health measures like water fluoridation and community-based prevention programs. These approaches should be complemented by individual-based strategies such as promotion of good oral hygiene practices, regular dental check-ups, and a diet low in sugar, as recommended by dental guidelines.”

**Table A4:** ChatGPT questions and answers about Pedodontics.

Type	Difficulty	Question	ChatGPT 3.5	ChatGPT 4
Binary	Easy	Is Orthopantomogram radiograph the ideal radiographic examination for detection of proximal caries in a 7-year-old patient?	No, an Orthopantomogram (OPG) radiograph is not considered the ideal radiographic examination for detection of proximal caries in a 7-year-old patient. OPGs provide a panoramic view of the entire mouth, but they have limitations in detecting small cavities or early stages of caries in between teeth. Bitewing radiographs are more suitable for detecting proximal caries in children, as they provide a more focused view of the teeth and can detect cavities at an early stage when they are easier to treat. The American Academy of Pediatric Dentistry recommends using bitewing radiographs at least once a year for children with a high risk of caries. Your dentist will determine the most appropriate radiographic examination based on your child’s individual needs.	“Orthopantomogram (OPG), also known as a panoramic radiograph, provides a broad view of the jaws, teeth, sinuses, nasal area, and temporomandibular joints (TMJs). It is useful for assessing the overall dental and maxillofacial health and development in children. However, it is not the most accurate or ideal radiographic examination for the detection of proximal caries, especially in a 7-year-old patient. According to the American Dental Association (ADA) and the American Academy of Pediatric Dentistry (AAPD), the following guidelines apply: 1. Bitewing Radiographs: Bitewing radiographs are the gold standard for diagnosing proximal (in-between) caries that are not clinically visible. They provide high resolution and detail that can help in detecting early caries lesions, even when they are underneath the contact point between two teeth. 2. Selective use of Radiographs: Radiographs should be taken only when necessary due to the potential risks associated with radiation exposure, especially in children. The ADA and AAPD recommend that the need for radiographs should be based on an individual caries risk assessment. For a 7-year-old, if the child is at low caries risk and there are no clinical indications (like pain or trauma), radiographs might not be necessary. On the other hand, if the child is at high risk for caries, posterior bitewings might be indicated at 6–12 month intervals. 3. Limitations of Panoramic Radiographs: While OPGs are useful for viewing the overall dental development, they have several limitations when it comes to diagnosing caries. They have lower resolution compared to bitewing or periapical radiographs, and they can be less accurate in detecting small caries lesions. Furthermore, they can result in image distortion and overlap, making it difficult to see proximal surfaces clearly. In conclusion, while panoramic radiographs have their uses, they are not the ideal choice for detecting proximal caries in a 7-year-old patient. Bitewing radiographs, used judiciously based on individual caries risk assessment, are the preferred choice. As always, the benefits and risks of radiographic examinations should be carefully considered, and the principle of ALARA (As Low As Reasonably Achievable) should be followed to minimize radiation exposure.”
	Medium	Is Nitrous oxide inhalation sedation contraindicated for pediatric dental patients who are diagnosed with asthma	Nitrous oxide inhalation sedation may be contraindicated for pediatric dental patients with a diagnosis of asthma. Although nitrous oxide is generally considered safe and effective for sedation in pediatric patients, it can cause respiratory depression and decrease oxygen saturation levels. Therefore, it is important for dentists to carefully evaluate each patient’s medical history and asthma control before administering nitrous oxide sedation. Alternative sedation methods may be recommended for patients with poorly controlled asthma to minimize the risk of respiratory complications. Your dentist will determine the most appropriate sedation option for your child based on their individual needs and medical history.	“Nitrous oxide, often referred to as “laughing gas,” is commonly used as a form of conscious sedation during dental procedures to help reduce anxiety and discomfort. According to the American Academy of Pediatric Dentistry (AAPD), as of my knowledge cutoff in September 2021, nitrous oxide inhalation sedation can be used in children with asthma, but with caution. Asthma is not generally considered an absolute contraindication for nitrous oxide sedation. However, if a child is having an active asthmatic attack, nitrous oxide should not be used. The AAPD’s “Guideline on Use of Nitrous Oxide for Pediatric Dental Patients” (revised 2018) states: “Nitrous oxide/oxygen may also be used with caution in patients with respiratory problems depending on the severity of the respiratory disease (e.g., asthma, cystic fibrosis).” The guidelines further recommend that the pediatric dentist should always take a thorough medical history and consult with the child’s physician if necessary. Some key considerations when using nitrous oxide in a child with asthma include: 1. Current Asthma Control: The child’s current asthma control and severity should be assessed. If a child’s asthma is well-controlled, nitrous oxide can be considered. If the child has severe or poorly controlled asthma, or is having an acute asthma attack, nitrous oxide should not be used. 2. Potential Triggers: Nitrous oxide is generally well-tolerated, but in some people, it can cause respiratory side effects such as coughing or bronchospasm, which could potentially trigger an asthma attack. The risk should be evaluated on an individual basis. 3. Emergency Preparedness: The dental team should be prepared to manage potential respiratory emergencies, including having a written emergency protocol and the necessary emergency equipment and medications available. In conclusion, while nitrous oxide can be used in children with asthma, it should be used with caution. The decision should be individualized based on the child’s current asthma control and severity, potential triggers, and the ability of the dental team to manage potential emergencies. Always consult with the child’s physician if there are any doubts or concerns.”
	Hard	Is One of the dental manifestations of biliary atresia green discoloration of the teeth?	Yes, green discoloration of the teeth is one of the dental manifestations of biliary atresia. Biliary atresia is a rare, congenital liver disease that affects the bile ducts, leading to a buildup of bile in the liver. The excess bile can cause discoloration of the teeth, giving them a greenish appearance. Other dental manifestations of biliary atresia may include delayed tooth eruption, enamel defects, and increased risk of dental caries. It is important for individuals with biliary atresia to receive regular dental care to maintain their oral health.	“Biliary atresia is a serious liver disorder that begins to affect newborns shortly after birth, wherein the bile ducts become inflamed, blocked, and scarred. Biliary atresia can lead to liver damage and cirrhosis of the liver, which is life-threatening if not treated. As per the medical and dental guidelines and literature up to my knowledge cutoff in September 2021, there is no direct correlation or evidence to suggest that biliary atresia causes a green discoloration of the teeth. Green discoloration of teeth can be caused by a variety of factors, including: 1. Poor oral hygiene and chromogenic bacteria: This can lead to green stain, especially on the cervical third of the tooth. 2. Certain medications and mouthwashes: Chlorhexidine and some other substances can cause tooth discoloration. 3. Biliverdin: In rare conditions, green discoloration could be caused by biliverdin, a green pigment present in bile. However, this is usually associated with conditions leading to hyperbilirubinemia, such as Rh incompatibility in newborns, and not typically seen in biliary atresia. 4. Extrinsic factors: These include dietary factors (certain foods, drinks, and candies), smoking, and occupational exposure to certain metals and substances. If a child with biliary atresia shows green discoloration of the teeth, it is important to investigate the cause, which could be unrelated to their liver condition. The child should be referred to a dentist for a thorough examination and appropriate treatment. It’s always recommended to keep regular dental check-ups to maintain oral health, especially for children dealing with serious health conditions, as some medications or conditions may have secondary effects on oral health.”
Descriptive	Easy	Mention the sequence of primary teeth eruption in the upper arch.	“The sequence of primary teeth eruption in the upper arch is typically as follows: 1. Central incisor (6–10 months) 2. Lateral incisor (7–12 months) 3. First molar (12–18 months) 4. Canine (16–22 months) 5. Second molar (20–30 months) It is important to note that the eruption times may vary slightly for each child and should be used as a general guideline. Your child’s dentist will be able to monitor their eruption pattern and address any concerns.”	“The eruption of primary teeth, also known as deciduous or baby teeth, follows a general pattern, although the exact timing can vary among individuals. According to the American Academy of Pediatric Dentistry (AAPD), the sequence of primary teeth eruption in the upper arch (maxilla) is as follows: 1. Central Incisors: The upper central incisors are typically the first to erupt, usually around 8–12 months of age. 2. Lateral Incisors: The upper lateral incisors are the next to come in, around 9–13 months of age. 3. First Molars: The upper first molars generally erupt next, usually around 13–19 months of age. 4. Canines: The upper canines, also known as cuspids, typically erupt between 16–22 months of age. 5. Second Molars: The upper second molars are typically the last to erupt, usually around 25–33 months of age. Remember, these are average ranges and some children may get their teeth earlier or later than these guidelines. Regular dental check-ups are important from an early age to ensure that tooth eruption is occurring as expected, and to detect and manage any potential dental problems.”
	Medium	What are the possible sequelae of palatally impacted canine if left un-treated?	“Possible sequelae of a palatally impacted canine, if left untreated, may include: 1. Malocclusion: The impacted canine can cause the other teeth to shift and become misaligned, affecting the bite and overall alignment of the teeth. 2. Damage to adjacent teeth: The impacted canine may cause damage to the roots of the adjacent teeth, leading to tooth loss. 3. Cyst formation: A cyst may form around the impacted tooth, causing infection, swelling, and pain. 4. Delayed eruption of permanent teeth: If the impacted canine is not treated, it can delay the eruption of permanent teeth in that area. 5. Aesthetic concerns: An impacted canine can cause visible bulging or swelling in the palate, affecting the appearance of the smile. It is important to seek treatment for a palatally impacted canine to prevent these potential complications.”	“A palatally impacted canine is a condition where the canine tooth, instead of erupting normally, becomes impacted (stuck) on the palatal (roof of the mouth) side of the dental arch. If left untreated, this condition can lead to several complications, as per the American Association of Endodontists (AAE) and the American Association of Oral and Maxillofacial Surgeons (AAOMS), these can include: 1. Resorption of Adjacent Teeth: The impacted canine can exert pressure on the roots of adjacent teeth, leading to root resorption. This is particularly a concern for the lateral incisors, which are located next to the canines. 2. Formation of Cysts or Tumors: Over time, a follicular cyst or tumor can develop around the crown of an impacted tooth, leading to bone destruction and displacement of adjacent teeth. This is a rare but serious complication. 3. Infection: While less common, infection can occur around an impacted tooth, leading to pain and swelling. 4. Orthodontic Problems: Impacted canines can interfere with the alignment of the other teeth, leading to crowding, gaps, or other orthodontic problems. 5. Aesthetic Concerns: The canines play a significant role in facial aesthetics. An unerupted or missing canine can affect the appearance of the smile. 6. Alveolar Bone Loss: Prolonged impaction can lead to alveolar bone loss in the area of the impacted tooth. The decision to treat a palatally impacted canine should be individualized and based on factors such as the patient’s age, the position of the impacted tooth, the presence of symptoms or associated pathology, the effect on adjacent teeth, and the potential for successful orthodontic treatment. The treatment options can include surgical exposure and orthodontic alignment, autotransplantation, or in some cases, extraction. Regular dental check-ups and early detection are key to managing this condition. If a canine tooth is not erupting as expected, an orthodontic consultation should be considered by around the age of 10.”
	Hard	What are the most common intra-oral features noted in Down Syndrome patients?	“The most common intra-oral features noted in Down Syndrome patients may include: 1. A small and narrow palate 2. A protruding tongue 3. Delayed eruption or missing teeth 4. Malocclusion, such as an open bite or crossbite 5. Increased risk of periodontal disease 6. Enamel defects, such as hypodontia or microdontia 7. Tongue thrusting or bruxism (teeth grinding) 8. Mouth breathing due to nasal obstruction 9. Fissured or cracked lips 10. Delayed speech and language development. It is important for individuals with Down Syndrome to receive regular dental care to manage these oral health concerns.”	“Down Syndrome, also known as trisomy 21, is a genetic disorder caused by the presence of all or part of a third copy of chromosome 21. It is associated with physical growth delays, characteristic facial features, and mild to moderate intellectual disability. There are also several common intra-oral features associated with Down Syndrome. According to the American Academy of Pediatric Dentistry (AAPD) and the Special Care Dentistry Association (SCDA), these can include: 1. Delayed Tooth Eruption: The eruption of both primary and permanent teeth may be delayed in individuals with Down Syndrome, and the sequence of tooth eruption may also be irregular. 2. Missing Teeth: Hypodontia, or the congenital absence of one or more teeth, is more common in individuals with Down Syndrome. The most frequently missing teeth are the third molars, followed by the second premolars and maxillary lateral incisors. 3. Malocclusion: Individuals with Down Syndrome are more likely to have malocclusion. This includes open bite, crossbite, and Class III malocclusion (where the lower jaw protrudes or the upper jaw is receded). 4. Periodontal Disease: There is a high prevalence of periodontal disease in individuals with Down Syndrome, often starting at an earlier age compared to the general population. This is due to multiple factors including immune response alterations, poor oral hygiene, and anatomical features. 5. Macroglossia: Apparent macroglossia (an enlarged tongue) is common, often due to hypotonia and the smaller size of the oral cavity. This can cause the tongue to protrude and contribute to mouth breathing, drooling, and speech difficulties. 6. Craniofacial Abnormalities: These can include a smaller maxilla with narrow palate, flattened midface, and a relatively larger mandible. 7. Taurodontism: A condition where the body of the tooth is enlarged at the expense of the roots. This is more common in the molars of individuals with Down Syndrome. 8. Bruxism: The prevalence of bruxism (teeth grinding or clenching) is reported to be higher in individuals with Down Syndrome. It’s important to note that not all individuals with Down Syndrome will express all these dental features. Moreover, dental care for individuals with Down Syndrome might require special considerations due to other health conditions commonly associated with the syndrome, such as congenital heart disease. Regular dental check-ups, good oral hygiene practices, and preventive dental care are key to maintaining oral health in individuals with Down Syndrome.”

**Table A5:** ChatGPT questions and answers about Prosthodontics.

Type	Difficulty	Question	ChatGPT 3.5	ChatGPT 4
Binary	Easy	Do you need to survey study model before drawing RPD design?	Yes, it is recommended to survey study models before drawing a removable partial denture (RPD) design. Surveying is the process of evaluating the teeth and soft tissue structures for their support and retention potential in order to design an RPD that will fit and function properly. This process involves using a special instrument called a surveyor to measure the contours and undercuts of the teeth and soft tissue. By surveying the study models, the dentist can determine the ideal placement and design of clasps, rests, and other components of the RPD to ensure optimal fit and function.	“Yes, surveying a study model (also known as a diagnostic cast or dental cast) is a critical step in the design of a removable partial denture (RPD). According to the American College of Prosthodontists (ACP) and the Academy of Prosthodontics, surveying helps in determining the path of insertion and removal of the RPD, identifying the undercut areas, and planning the placement of key RPD components such as clasps and rests. The specific steps involved in surveying a study model for an RPD design include: 1. Identifying the Tilt: The model is mounted on a surveyor, a device that allows the model to be tilted in various directions. The surveyor has a analyzing rod, which is passed over the teeth to find the optimal path of insertion and removal that offers the best balance between retention and stability. 2. Marking the Survey Line: The survey line (also known as the height of contour or the surveyor’s line) is marked on the model. This is the line along which the clasp arms of the RPD will engage the teeth. 3. Identifying Undercuts: Undercut areas below the survey line are identified. These areas can be utilized for additional retention, or they may need to be blocked out (made less deep) if they interfere with the path of insertion and removal. 4. Planning the RPD Design: Based on the survey, the design of the RPD can be planned. This includes deciding on the types and positions of clasps, the design of the major and minor connectors, the placement of rests, and the incorporation of any prosthetic teeth. Therefore, surveying a study model is an essential step in the design of an RPD. It helps to ensure that the final RPD will have optimal fit, function, and aesthetics, and that it will be as comfortable as possible for the patient.”
	Medium	Neutral zone impression techniques is used in highly atrophic maxillary ridge.	Yes, neutral zone impression techniques can be used in highly atrophic maxillary ridges. This technique involves creating an impression of the oral tissues in the neutral zone, which is the area where the forces from the cheeks and tongue are balanced. This can help to achieve better retention and stability for a removable prosthesis in cases where there is significant bone resorption. The neutral zone can also help to improve speech and comfort for the patient. However, this technique may require specialized training and equipment, and the dentist will determine if it is the most appropriate option for a patient with a highly atrophic maxillary ridge.	“The neutral zone is the area in the oral cavity where the forces of the tongue pressing outwards are neutralized by the forces of the cheeks and lips pressing inwards. In other words, it’s a region of equilibrium where the forces exerted by the muscles are minimized. The concept of the neutral zone is particularly relevant in the fabrication of dentures for patients with severely atrophic (resorbed) ridges or unusual muscle attachments. Here are the general steps of the neutral zone impression technique, as outlined by the American College of Prosthodontists (ACP) and the Academy of Prosthodontics: 1. Preliminary Impressions and Casts: Preliminary impressions of the maxillary and mandibular arches are made using an appropriate material such as alginate. These impressions are used to fabricate diagnostic casts. 2. Record Base and Wax Rim Fabrication: A record base and occlusion rim are fabricated on the diagnostic casts. Modifications may be necessary to account for the atrophic ridge. 3. Neutral Zone Recording: The patient is asked to perform various oral movements such as swallowing, speaking certain words, smiling, and pursing the lips. A low fusing compound or soft wax is added to the occlusion rim and shaped by these functional movements, recording the neutral zone. This process may be repeated several times to ensure an accurate recording. 4. Jaw Relation Records and Try-in: The neutral zone recording is used to help establish the proper jaw relation records and tooth arrangement. A try-in of the denture setup in wax is performed to verify the accuracy of the records and the esthetics and function of the denture. 5. Denture Fabrication: The denture is processed, finished, and polished. The contours of the denture should reflect the shape of the neutral zone to help optimize denture stability and function. 6. Denture Insertion and Adjustment: The denture is inserted and checked for fit, occlusion, and stability. Adjustments are made as necessary. It’s important to note that the neutral zone technique requires experience and skill to execute effectively. It may not be suitable for all patients with atrophic ridges and should be used in conjunction with a thorough patient assessment and other appropriate prosthodontic techniques.”
	Hard	Dahl concept can be used in localised anterior teeth wear and no space available in RCP. Do you agree with previous statement.	Yes, the Dahl concept can be used in cases of localized anterior teeth wear and no space available in the rest position (RCP). The Dahl concept involves restoring the vertical dimension of occlusion by selectively grinding down the posterior teeth, creating space for the anterior teeth to move into a more stable and esthetic position. This technique can be effective in cases where there is localized anterior teeth wear and limited space available for restorative treatment. However, it is important for the dentist to carefully evaluate each case and determine if the Dahl concept is the most appropriate treatment option for the patient.	“Yes, I agree with the statement. The Dahl concept is a conservative treatment approach designed to manage cases of localized anterior tooth wear where there is insufficient space for restoration in the Retruded Contact Position (RCP, also known as centric relation). According to the British Society for Restorative Dentistry (BSRD) and the British Society of Prosthodontics, the Dahl concept involves creating a “therapeutic space” by selectively adjusting the occlusion. It makes use of the body’s natural adaptive capacity to generate space for restorations. The Dahl concept generally involves these steps: 1. Assessment and Diagnosis: A comprehensive assessment is performed, including the examination of the patient’s occlusion, oral hygiene status, and the extent and pattern of tooth wear. 2. Treatment Planning: A treatment plan is formulated, taking into account the patient’s functional and aesthetic needs. Diagnostic wax-ups and/or a diagnostic set-up in an articulator may be used to plan the treatment. 3. Dahl Appliance: An intraoral appliance, often a partial coverage splint or indirect restorations on the worn teeth, with an increased occlusal vertical dimension (i.e., “raised bite”) is placed. This appliance contacts the opposing teeth in the region of tooth wear, resulting in disclusion (i.e., no contact) of the posterior teeth. 4. Adaptation Period: Over a period of weeks to months, the posterior teeth will supererupt (move towards the opposing teeth) until they reestablish contact with the opposing arch. This generates the necessary space to restore the worn anterior teeth without changing the overall vertical dimension of occlusion. 5. Restoration: Once the therapeutic space is achieved, the worn anterior teeth can be restored, typically with adhesive, minimally invasive restorations. The Dahl concept is a useful tool in the management of localized anterior tooth wear. However, it requires careful case selection, accurate diagnosis, and meticulous treatment planning and execution. It’s also essential to monitor these cases closely during the adaptation period and after the final restorations are placed. This approach may not be suitable for all patients and other treatment options should also be considered based on the patient’s specific condition and needs.”
Descriptive	Easy	what are the clinical stages of complete denture construction?	“The clinical stages of complete denture construction typically include: 1. Preliminary impressions: The first step is to take impressions of the patient’s mouth to create a custom tray that fits their gums and surrounding tissues. 2. Final impressions: Using the custom tray, the dentist will take final impressions of the patient’s mouth to accurately capture the shape and size of their gums and surrounding tissues. 3. Bite registration: The dentist will record the patient’s bite to determine the proper relationship between the upper and lower jaws. 4. Try-in: A wax model of the denture is created for the patient to try in their mouth. Any necessary adjustments are made at this stage. 5. Processing: Once the try-in is approved, the wax model is processed into a final denture using heat and pressure. 6. Delivery: The final denture is delivered to the patient, and any final adjustments are made for comfort and fit. It is important to note that the specific stages and techniques may vary depending on the individual needs of the patient and the dentist’s preferences.”	“The fabrication of complete dentures is a detailed and multi-step process. Although the steps may vary slightly depending on the dentist’s preference and patient’s specific needs, the basic clinical stages as recommended by the American College of Prosthodontists (ACP) and the Academy of Prosthodontics are as follows: 1. Preliminary Impressions: This involves taking an initial impression of the maxillary and mandibular edentulous arches using a stock tray and an appropriate impression material, usually alginate. This impression is used to fabricate a custom impression tray. 2. Final Impressions: A custom tray is used to make a more accurate final impression of the edentulous ridges. The material used for this impression can be a higher viscosity alginate, polyvinyl siloxane, or polyether, among others. 3. Occlusal Rim Fabrication: Record bases are constructed on the final cast and wax occlusal rims are attached. These are used to record the maxillomandibular relationship and to aid in the selection and arrangement of denture teeth. 4. Jaw Relation Records: The vertical dimension of occlusion (VDO) and centric relation (CR) are recorded. These records are used to articulate the casts in the correct relationship on a dental articulator. 5. Selection of Denture Teeth: The shape, size, and color of the artificial teeth are selected based on the patient’s facial features, oral structures, and personal preferences. 6. Try-in of Denture Teeth: The teeth are set in wax and tried in the patient’s mouth to verify aesthetics, phonetics, occlusion, and the maxillomandibular relationship. Adjustments are made as needed. 7. Denture Processing: Once the try-in is approved, the waxed denture is processed into a final product. This involves replacing the wax with a hard, durable acrylic resin. 8. Denture Insertion: The completed dentures are inserted and checked for fit, stability, and occlusion. Adjustments are made as necessary. 9. Post-insertion Adjustments: The patient is scheduled for follow-up appointments to manage any post-insertion issues such as sore spots or difficulties with chewing or speaking. 10. Recall and Maintenance: Regular recall appointments are scheduled to monitor the patient’s adaptation to the dentures, check for changes in oral tissues, and provide professional cleaning of the dentures. Each stage requires careful attention to detail to ensure a well-fitting, functional, and aesthetically pleasing result. Patient education and communication are key throughout the process to ensure patient satisfaction with the final outcome.”
	Medium	What are the uses of face-bow?	“A face-bow is a dental instrument used to record the position of the patient’s jaws in relation to their temporomandibular joints. It is an essential tool in creating accurate and well-fitting prosthodontic restorations. Some of the uses of a face-bow include: 1. Determining the correct orientation of the maxillary arch in relation to the temporomandibular joints for accurate mounting of casts on an articulator. 2. Recording the patient’s bite and jaw movements to create a more precise occlusal relationship. 3. Evaluating the position of the maxillary arch in relation to the facial midline. 4. Assessing the patient’s occlusion and making necessary adjustments to ensure proper function and comfort. The use of a face-bow can help to improve the overall success and longevity of prosthodontic restorations.”	“A face-bow is a dental instrument used in prosthodontics and orthodontics. It helps to record the spatial relationship between the upper jaw (maxilla) and the temporomandibular joint (TMJ), and to transfer this relationship accurately to an articulator, a device that simulates the movements of the jaws. According to the American College of Prosthodontists (ACP) and the Academy of Prosthodontics, the specific uses of a face-bow include: 1. Transferring Maxillary Cast: It helps to accurately mount the maxillary (upper) cast on the dental articulator in the same anatomical orientation as in the patient’s mouth. 2. Articulator Adjustment: It allows for the adjustment of the articulator to mimic the patient’s jaw movements as closely as possible. This is crucial for the accurate reproduction of the patient’s occlusion and function. 3. Prosthetic Design: It assists in the design and fabrication of dental prostheses such as crowns, bridges, and dentures. By accurately replicating the patient’s jaw relationships and movements, it helps to ensure that the prostheses will fit and function correctly. 4. Orthodontic Treatment Planning: In orthodontics, a face-bow can be used to help analyze and plan treatment for malocclusions and other jaw-related issues. 5. Diagnosis and Treatment Planning: The face-bow can also assist in diagnosing temporomandibular disorders or other occlusal issues and planning their treatment. 6. Communication: It aids in communication among dental professionals by providing a standardized reference of the patient’s occlusion. While the use of a face-bow can enhance the precision of dental treatment, its use may not be necessary in all cases. According to the American Dental Association (ADA) and the ACP, the decision to use a face-bow should be based on the complexity of the case, the specific needs of the patient, and the judgement of the dental professional. Cases that involve major changes to the occlusion, full-mouth rehabilitation, or complex prosthodontic treatment often benefit from the use of a face-bow.”
	Hard	what is shortened dental arch concept ?	The shortened dental arch (SDA) concept is a treatment approach that advocates for preserving a functional dentition by avoiding the need for extractions and maintaining a reduced number of posterior teeth. This concept is based on the belief that a reduced number of teeth can still provide adequate masticatory function and esthetics, as long as the remaining teeth are properly positioned and occlude with stability. The SDA concept is often used in older patients or those with limited dental resources and focuses on retaining the teeth that are essential for maintaining oral function and esthetics. Some studies have shown that the SDA concept can be a successful and cost-effective treatment option, but it may not be suitable for all patients and requires careful evaluation and planning by the dentist.	“The Shortened Dental Arch (SDA) concept is a treatment approach in prosthodontics that suggests maintaining or restoring a certain minimum number of occluding pairs of teeth, specifically the anterior and premolar regions, while not necessarily replacing the molar teeth. The concept was introduced by Dutch prosthodontist Professor Eijkman and his colleagues, and was further developed by Professor Kayser. The SDA concept assumes that a dental arch with at least all incisors and canines, plus the first premolars, provides sufficient function. This generally means having at least 10 occluding pairs of teeth. According to the World Dental Federation (FDI) and the American College of Prosthodontists (ACP), the SDA concept has been used in the following scenarios: 1. To avoid extensive prosthodontic treatments: The SDA concept can be an alternative to extensive prosthodontic treatments such as complete arch fixed or removable prostheses. 2. Treatment of periodontally compromised patients: In patients with advanced periodontal disease, maintaining a shortened dental arch can help to preserve the remaining teeth and stabilize the periodontal condition. 3. Treatment of elderly or medically compromised patients: In elderly or medically compromised patients, the SDA concept can provide a functional and less complex prosthodontic solution. 4. Financial considerations: The SDA concept can also be a more affordable option for patients who cannot afford more extensive prosthodontic treatments. Several long-term studies have shown that patients with a shortened dental arch can have satisfactory oral function, masticatory ability, and oral health-related quality of life. However, it’s important to note that the SDA concept is not suitable for all patients, and its use should be based on a thorough clinical examination, patient’s specific needs and preferences, and the clinician’s judgement. If the patient experiences functional problems or is not satisfied with the SDA, additional molar replacement can be considered. Regular follow-up and maintenance are important for patients treated according to the SDA concept.”

**Table A6:** ChatGPT questions and answers about Orthodontics.

Type	Difficulty	Question	ChatGPT 3.5	ChatGPT 4
Binary	Easy	Is the primate space located between the Mandibular canine and lateral incisor?	No, the primate space is not located between the mandibular canine and lateral incisor. The primate space refers to the natural space between the primary canine and first primary molar in the upper and lower jaws. This space provides room for the permanent teeth to erupt into the mouth. In some cases, a primate space may also be present between the lateral incisor and canine, but this is not considered the typical location.	“No, the primate space is not located between the mandibular canine and lateral incisor. The term “primate space” refers to the physiological spaces present in the primary (or deciduous) dentition. According to the American Academy of Pediatric Dentistry (AAPD), these spaces are generally located: 1. In the maxilla (upper jaw), between the lateral incisor and the canine. 2. In the mandible (lower jaw), between the canine and the first primary molar. These spaces play a crucial role in the development of the dentition. They help to accommodate the larger permanent teeth that will later erupt and ensure there is sufficient space for their proper alignment. If these spaces close prematurely or are not present, it may indicate future crowding or malalignment of the permanent teeth. Therefore, it is important for dental practitioners to monitor these spaces during a child’s oral health assessments. If there are concerns about the development of a child’s dental arches or the alignment of the teeth, a consultation with an orthodontist may be recommended.”
	Medium	Is Cephalometrics required for all orthodontic assessment?	No, cephalometrics is not required for all orthodontic assessments. Cephalometric analysis involves taking a radiograph of the side of the head and measuring specific angles and distances to evaluate the relationship between the jaws and teeth. While this can be a helpful tool in creating a treatment plan and monitoring progress, it is not always necessary. Other factors, such as clinical examination, dental models, and photographs, can also provide valuable information for orthodontic assessment. Your orthodontist will determine if cephalometrics is necessary for your specific case.	“Cephalometric radiographs are a common diagnostic tool used in orthodontics. They provide a side (lateral) view of the face and skull and are used to assess the anatomical relationships among various skeletal and dental structures. These radiographs can help in the diagnosis of orthodontic problems, planning treatment, and monitoring progress. However, according to guidelines by the American Association of Orthodontists (AAO) and the American Dental Association (ADA), a cephalometric radiograph is not always required for every orthodontic assessment. The decision to take a cephalometric radiograph should be based on the individual needs of the patient, the type and complexity of the orthodontic problem, and the planned treatment approach. For complex cases, such as those involving significant skeletal discrepancies, potential growth modification, orthognathic surgery planning, or complex mechanotherapy, cephalometric radiographs can provide valuable information that may not be obtainable from a clinical examination alone. However, for simpler cases, such as minor tooth movement or the treatment of simple malocclusions, a cephalometric radiograph may not be necessary. The treating orthodontist can often make a diagnosis and treatment plan based on a comprehensive clinical examination, study models of the teeth, and standard dental radiographs. The ADA and AAO emphasize that radiographs should only be taken when there is an expectation that the diagnostic yield will affect patient care. The benefits of taking the radiograph should outweigh the risks of radiation exposure. This aligns with the principle of “as low as reasonably achievable” (ALARA) in radiography, which aims to minimize radiation exposure to patients. In summary, while cephalometric radiographs are a valuable tool in orthodontics, whether they are needed should be determined on a case-by-case basis. The orthodontist should explain the reasons for any proposed radiographs to the patient (or their guardian), and obtain informed consent before proceeding.”
	Hard	Is stainless steel the best material choice for patients with a nickel allergy?	No, stainless steel is not the best material choice for patients with a nickel allergy. Stainless steel contains a significant amount of nickel, which can cause allergic reactions in some individuals. For patients with a known nickel allergy, alternative materials such as titanium or ceramic can be used for orthodontic appliances. It is important for patients to inform their orthodontist of any known allergies to ensure the use of appropriate materials.	“Stainless steel is a commonly used material in dentistry due to its durability and strength. However, it contains nickel, a metal that can cause allergic reactions in some people. Nickel is present in a significant percentage in stainless steel (usually between 8% and 12% in dental stainless steel), and therefore may not be the best choice for patients with a known nickel allergy. The American Dental Association (ADA) and the American Association of Orthodontists (AAO) guidelines advise that alternative materials should be considered for patients with a known nickel allergy. These might include: 1. Titanium: Titanium and titanium alloys are highly biocompatible and generally well-tolerated, making them a good option for patients with metal allergies. 2. Ceramic: Ceramic materials, such as zirconia or alumina, are often used for crowns, bridges, and orthodontic brackets. They are an excellent choice for patients with metal allergies, and they also provide excellent aesthetics due to their tooth-like color. 3. Polymer materials: Certain types of plastic or composite materials may be used, particularly for orthodontic retainers and other removable appliances. 4. Gold: Gold and its alloys have been used in dentistry for many years and are highly biocompatible, but they’re not commonly used in orthodontics due to their color and cost. Before treatment, a thorough medical and dental history should be obtained to identify any known allergies. If a patient has a suspected or confirmed nickel allergy, the dentist or orthodontist should discuss the available material options and their pros and cons with the patient, and choose the most suitable one based on the patient’s specific needs and preferences. In cases of doubt, referral to an allergist for testing might be indicated. It’s also important to note that allergies can develop over time, so any changes in patient’s medical history should be updated regularly.”
Descriptive	Easy	What are the potential risks for orthodontic treatment?	“Some potential risks for orthodontic treatment may include: 1. Tooth decay or gum disease due to difficulty maintaining proper oral hygiene with braces. 2. Allergic reactions to materials used in orthodontic appliances. 3. Root resorption, which is a shortening of the roots of teeth due to pressure from braces. 4. Soft tissue irritation or sores in the mouth from the brackets and wires. 5. Relapse, where teeth may shift back to their original position after treatment. 6. Temporomandibular joint (TMJ) pain or dysfunction. It is important to discuss any potential risks and concerns with your orthodontist before starting treatment. They will also provide instructions and tips to help minimize these risks and ensure a successful treatment outcome.”	“Orthodontic treatment, like any other dental or medical treatment, comes with potential risks. The American Association of Orthodontists (AAO) and the American Dental Association (ADA) outline several potential risks, and it’s important that the orthodontist discusses these with the patient (or the patient’s guardian) before the start of treatment: 1. Root Resorption: During orthodontic treatment, slight shortening of roots (root resorption) can occur and is usually of no clinical consequence. However, on rare occasions, greater amounts of resorption can occur. 2. Decalcification and Tooth Decay: Braces and other orthodontic appliances can make oral hygiene more challenging. This can lead to decalcification (white spot lesions), cavities, or gum disease if the patient does not maintain good oral hygiene. 3. Periodontal Disease: If not properly cleaned, orthodontic bands and brackets can lead to increased plaque and tartar buildup, which can result in gum inflammation and periodontal disease. 4. Relapse: Teeth may change position after removal of braces (relapse) if retainers are not worn as directed. 5. Oral Tissue Irritation: Brackets, wires, and other appliances can irritate the cheeks, lips, and gums, especially when they are first applied or adjusted. 6. Allergic Reactions: Some patients may have allergic reactions to latex (used in elastics) or metal (used in braces and wires). 7. TMJ Disorders: Some patients might experience temporomandibular joint (TMJ) pain or dysfunction during or after orthodontic treatment. However, the relationship between orthodontics and TMJ disorders is not fully understood and is currently a topic of research. 8. Injury from Orthodontic Appliances: In rare cases, orthodontic appliances may be accidentally swallowed, aspirated, or cause injury to the teeth or oral tissues, especially in patients who engage in contact sports. 9. Unfavorable Growth: Unpredictable changes in growth can affect the outcome of orthodontic treatment. It’s important to note that these risks can be minimized by following the orthodontist’s instructions regarding appliance care, oral hygiene, diet, and retainer wear. Regular dental checkups and cleaning are also crucial during orthodontic treatment. The orthodontist should explain these potential risks to the patient and take informed consent before starting treatment. The benefits of orthodontic treatment usually far outweigh the potential risks, as it can improve oral function, aesthetics, and overall oral health.”
	Medium	when does eruptive tooth movement begins?	Eruptive tooth movement typically begins around the age of six months, when the primary teeth start to emerge. The first teeth to erupt are usually the lower central incisors, followed by the upper central incisors. The sequence of eruption can vary slightly from child to child, but most primary teeth should be present by the age of three. Eruptive tooth movement continues throughout childhood and into early adulthood as the permanent teeth replace the primary teeth.	“Eruptive tooth movement is the process by which a tooth moves from its developmental position within the jaws to its functional position in the oral cavity. This movement begins before the tooth has fully formed and continues until the tooth has reached its final position in the occlusal plane. According to the American Academy of Pediatric Dentistry (AAPD) and the American Dental Association (ADA), the timeline for tooth eruption varies, but here’s a general guideline: 1. Primary Teeth (Deciduous or “Baby” Teeth): The process of eruption for primary teeth usually begins around 6 to 10 months of age, with the lower central incisors typically erupting first. By the age of 3, most children have a full set of 20 primary teeth. 2. Permanent Teeth: The first permanent teeth to erupt are usually the first molars and lower central incisors, which typically occur around 6 to 7 years of age. The process continues until approximately 21 years of age, ending with the eruption of the third molars (wisdom teeth), if present. It’s important to note that there is a wide range of normal variability in the timing of tooth eruption. Some children may get teeth earlier or later than the average. Regular dental check-ups are important during childhood to monitor the progress of tooth eruption and to identify any potential problems, such as delayed eruption, early tooth loss, or crowding, which could require intervention. If a tooth has not erupted when expected, the dentist or orthodontist may order radiographs to check the position and development of the tooth and determine whether any treatment is needed. If the tooth is not going to erupt on its own, a procedure to assist its eruption may be necessary. Parents should be informed about the general timeline for tooth eruption and advised to contact their dentist if they have any concerns about their child’s dental development.”
	Hard	When is orthognathic surgery indicated?	Orthognathic surgery, also known as corrective jaw surgery, is indicated for individuals with severe jaw misalignment or discrepancies that cannot be corrected with orthodontic treatment alone. It may also be recommended for individuals with facial trauma or congenital disorders affecting the jaw. Orthognathic surgery can help improve the function and appearance of the jaw and may be necessary to correct issues such as difficulty chewing, breathing, or speaking, facial asymmetry, and temporomandibular joint (TMJ) disorders. This type of surgery is typically performed during late adolescence or early adulthood when the jaw has finished growing.	“Orthognathic surgery, also known as corrective jaw surgery, is performed to correct a wide range of minor and major skeletal and dental irregularities, including the misalignment of jaws and teeth. It can improve chewing, speaking, and breathing, and, in some cases, enhance appearance. According to the American Association of Oral and Maxillofacial Surgeons (AAOMS) and the American Association of Orthodontists (AAO), orthognathic surgery is considered when: 1. Severe Malocclusion: Orthognathic surgery may be indicated when there’s a severe malocclusion (incorrect bite) that can’t be corrected with orthodontic treatment alone. 2. Facial Asymmetry: Significant facial asymmetry, such as noticeable differences between the two sides of the face, can be addressed with this surgery. 3. Sleep Apnea and Breathing Problems: For some patients with obstructive sleep apnea or difficulty breathing due to skeletal issues, orthognathic surgery can help open the airway and improve breathing. 4. Temporomandibular Joint (TMJ) Disorders: If conservative treatments are unsuccessful, orthognathic surgery may be considered for certain TMJ disorders, particularly if the condition is related to a structural issue with the jaw. 5. Congenital Conditions: Conditions present at birth, such as cleft palate or certain craniofacial syndromes, often require orthognathic surgery as part of their comprehensive treatment. 6. Inability to Close the Lips Comfortably: If a person cannot close their lips comfortably without straining, surgery may be considered. Before surgery, most patients will undergo orthodontic treatment to move the teeth into a new position. Post-surgery, further orthodontic treatment is often needed to fine-tune the occlusion. The decision to proceed with orthognathic surgery should be made after a thorough evaluation and discussion of the benefits, risks, and alternatives. The surgical plan should be tailored to the individual patient’s needs and goals, and informed consent should be obtained. It’s important to note that orthognathic surgery is typically considered a last resort when less invasive treatments are unlikely to achieve the desired results. The decision to proceed with surgery should be made in collaboration with a team of healthcare providers, including an oral and maxillofacial surgeon, orthodontist, and other medical and dental specialists as needed.”

**Table A7:** ChatGPT questions and answers about Surgery.

Type	Difficulty	Question	ChatGPT 3.5	ChatGPT 4
Binary	Easy	Is cleft lip and palate (CLP) the most common congential deformity in the craniofacial region?	Yes, cleft lip and palate (CLP) is one of the most common congenital deformities in the craniofacial region, affecting approximately one in every 700–1000 live births worldwide. It is a birth defect where there is an opening or split in the lip and/or palate, caused by incomplete fusion of the facial structures during fetal development. This condition can vary in severity, and treatment may involve surgical repair, orthodontic and prosthodontic treatment, and speech therapy. Early intervention and a multidisciplinary team approach are essential for successful treatment of cleft lip and palate.	“Yes, according to the Centers for Disease Control and Prevention (CDC), the American Cleft Palate-Craniofacial Association (ACPA), and the American Dental Association (ADA), cleft lip and palate (CLP) are among the most common congenital deformities in the craniofacial region. Cleft lip and cleft palate occur when a baby’s lip or mouth does not form properly during pregnancy. These orofacial clefts are divided into two main types: 1. Cleft Lip (with or without Cleft Palate): This type of cleft results in a physical split or separation of the two sides of the upper lip and appears as a narrow opening or gap in the skin of the upper lip. This separation often extends beyond the base of the nose and includes the bones of the upper jaw and/or upper gum. 2. Cleft Palate: A cleft palate is a split or opening in the roof of the mouth (palate), and can involve the hard palate and/or the soft palate. The occurrence of orofacial clefts varies among different ethnic groups and geographical areas. In the United States, according to the CDC, cleft lip and palate occur in about 1 in every 1600 births, and cleft palate alone (without cleft lip) occurs in about 1 in every 1700 births. Treatment for cleft lip and cleft palate often involves a team approach, with various healthcare providers such as a plastic surgeon, oral and maxillofacial surgeon, orthodontist, pediatric dentist, speech pathologist, audiologist, and others working together to provide comprehensive care. The treatment plan is individualized for each child and typically includes surgical repair, orthodontic treatment, speech therapy, and other supportive treatments as needed. It’s important for parents to know that with the appropriate treatment plan, most children with cleft lip and palate can achieve normal appearance, speech, and quality of life.”
	Medium	Does the first molar carry the highest risk of fracture during extraction?	Yes, the first molar is often considered the tooth with the highest risk of fracture during extraction. This is due to its size, shape, and position in the mouth, making it more difficult to remove compared to other teeth. Additionally, the roots of the first molar are often fused and curved, requiring more force and skill to extract. To minimize the risk of fracture, dentists may use specialized techniques and instruments, such as elevators and forceps, to carefully and gently remove the tooth. In some cases, the tooth may need to be sectioned into smaller pieces for safer extraction.	“The risk of tooth fracture during extraction is not solely dependent on the type of tooth, but on a variety of factors. According to the American Dental Association (ADA) and the American Association of Oral and Maxillofacial Surgeons (AAOMS), these factors include: 1. Tooth morphology: The structure and form of the tooth can affect the risk of fracture. Teeth with large restorations, extensive caries, or previous root canal treatment may have compromised structural integrity and thus higher risk of fracture during extraction. 2. Root morphology: Teeth with multiple roots, curved roots, or fused roots can pose a greater challenge during extraction and increase the risk of fracture. 3. Bone density and quality: Dense bone, often found in the mandible (lower jaw), can make extraction more difficult and increase the risk of tooth or bone fracture. 4. Patient age: Older patients often have more brittle teeth and denser bone, both of which can increase the risk of fracture during extraction. 5. Operator skill and technique: The dentist’s or surgeon’s experience, skill, and technique play a significant role in the risk of tooth fracture. While it is not accurate to state that the first molar carries the highest risk of fracture, lower molars, in general, can be more challenging to extract due to their two roots that can be quite divergent. Upper first molars, with three roots, can also be challenging, particularly when the roots are fused or curved. The risk of tooth fracture, as well as other potential complications, should be discussed with the patient before the extraction procedure. If a tooth is deemed to have a high risk of complications, the extraction should be performed by a dentist or surgeon with the appropriate level of experience and skill. In some cases, a referral to an oral and maxillofacial surgeon may be indicated.”
	Hard	Is epinephrine in local anesthesia contraindicated for patients with cardiac conditions?	Yes, epinephrine in local anesthesia may be contraindicated for patients with certain cardiac conditions. Epinephrine, also known as adrenaline, is a vasoconstrictor that is commonly added to local anesthetics to prolong their effects and minimize bleeding during dental procedures. However, for patients with certain cardiac conditions, such as uncontrolled hypertension or a history of heart attack or stroke, the use of epinephrine may increase the risk of adverse effects, such as increased heart rate and blood pressure. In these cases, alternative anesthetic options may be recommended by the dentist to ensure the safety and well-being of the patient.	“Epinephrine, also known as adrenaline, is often added to local anesthetics in dental procedures to prolong the duration of the anesthetic effect and to reduce systemic absorption, thereby minimizing toxicity. However, epinephrine can also increase heart rate and blood pressure, which may be a concern for patients with certain cardiovascular conditions. According to the American Dental Association (ADA) and the American Heart Association (AHA), the use of local anesthetics with epinephrine is generally safe for most patients with cardiovascular disease, provided that certain precautions are taken: 1. Limited Dosage: The dosage of epinephrine should be kept as low as possible. The ADA recommends a maximum dose of 0.04 mg of epinephrine for patients with significant cardiovascular disease. 2. Aspiration: Aspiration prior to injection is recommended to avoid intravascular injection, which could result in a rapid increase in heart rate and blood pressure. 3. Slow Administration: The anesthetic should be administered slowly to minimize the risk of intravascular injection and to reduce the peak plasma concentration of the anesthetic. 4. Monitor Patient: The patient’s vital signs should be monitored during the procedure. For some patients with severe cardiovascular disease, such as those with unstable angina or uncontrolled high blood pressure, consultation with their cardiologist may be indicated before proceeding with dental procedures that require local anesthesia with epinephrine. It’s important to note that the stress and pain of dental procedures can also increase heart rate and blood pressure, potentially posing a greater risk than the use of epinephrine in local anesthesia. Therefore, adequate pain control is crucial and the benefits of using local anesthesia with epinephrine often outweigh the risks. As with any medical procedure, the dentist should discuss the benefits and potential risks with the patient (or the patient’s guardian) and obtain informed consent before administering local anesthesia with epinephrine.”
Descriptive	Easy	What are the treatement protocol for dry sockets?	“The treatment protocol for dry sockets, also known as alveolar osteitis, may include: 1. Irrigation of the socket: The dentist may use a syringe to gently flush out the socket with a saline or antiseptic solution to remove any debris and promote healing. 2. Dressing placement: A medicated dressing, such as eugenol or zinc oxide, may be placed in the socket to relieve pain and promote healing. 3. Pain management: The dentist may prescribe pain medication or recommend over-the-counter pain relievers to help manage discomfort. 4. Antibiotics: If there is evidence of infection, the dentist may prescribe antibiotics to prevent further complications. 5. Follow-up appointments: The patient will be scheduled for follow-up appointments to monitor healing and ensure proper treatment. It is important to note that the treatment protocol may vary depending on the severity of the dry socket and the individual needs of the patient.”	“Dry socket, or alveolar osteitis, is a potential complication that can occur after a tooth extraction, particularly after removal of wisdom teeth. It happens when the blood clot at the extraction site is dislodged or dissolves before the wound has healed, exposing the underlying bone and nerves, and causing intense pain. According to the American Dental Association (ADA) and the American Association of Oral and Maxillofacial Surgeons (AAOMS), the treatment protocol for dry socket typically includes the following steps: 1. Irrigation: The socket is gently irrigated to remove any debris that may be contributing to the patient’s discomfort. 2. Medicated Dressing: A medicated dressing or packing is often placed in the socket to help relieve pain. This dressing usually contains a local anesthetic for pain relief, and may contain other medications to reduce inflammation and promote healing. The dressing may need to be replaced every few days in the initial stages of treatment. 3. Pain Management: Over-the-counter pain relievers, such as ibuprofen, can be used for pain control. In some cases, stronger prescription pain medications may be needed. 4. Antibiotics: If there are signs of infection (which is different from dry socket), antibiotics may be prescribed. 5. Home Care Instructions: Patients should be given instructions for home care, which may include rinsing with warm salt water, avoiding smoking or the use of straws (which can dislodge the clot), and consuming soft foods. Good oral hygiene is important, but the patient should avoid the extraction site when brushing and flossing the other teeth. 6. Follow-Up Appointments: The patient should be seen for follow-up visits until the dry socket has healed. This allows the clinician to monitor the healing process, replace the medicated dressing as needed, and manage the patient’s pain. Prevention is a crucial part of managing dry socket. Risk factors for dry socket include smoking, oral contraceptives, and a history of dry socket after previous extractions. Patients should be informed about these risk factors and given instructions on how to reduce their risk. If a patient is at high risk for dry socket, the dentist may consider placing a medicated dressing at the time of extraction as a preventive measure.”
	Medium	When is prohlactic antibiotics recommended before extraction?	Prophylactic antibiotics, also known as premedication, may be recommended before tooth extraction in patients with certain medical conditions or a history of complications. These may include patients with a weakened immune system, heart conditions, or a history of joint replacement surgery. Premedication helps to prevent infection and other complications that may arise during or after the extraction procedure. The decision to prescribe prophylactic antibiotics is based on the individual patient’s medical history and the dentist’s professional judgement. It is important for patients to inform their dentist of any relevant medical conditions and medications before undergoing a tooth extraction.	“Prophylactic antibiotics are used to help prevent infections in certain patients before they undergo invasive procedures, including tooth extractions. The guidelines for prescribing prophylactic antibiotics before dental procedures are outlined by the American Dental Association (ADA) and the American Heart Association (AHA). According to these guidelines, prophylactic antibiotics are recommended before dental procedures for patients with the following conditions: 1. Artificial Heart Valves: Patients with prosthetic cardiac valves are at an increased risk of infective endocarditis, a serious infection of the inner lining of the heart chambers and heart valves. 2. History of Infective Endocarditis: Patients who have had infective endocarditis in the past are at a higher risk of getting it again. 3. Certain Congenital Heart Conditions: These include cyanotic congenital heart disease that has not been fully repaired, a congenital heart defect that’s been completely repaired with prosthetic material or a device for the first six months after the repair procedure, or any repaired congenital heart defect with residual defects. 4. Cardiac Transplant Recipients with Valve Regurgitation due to a Structurally Abnormal Valve: These patients are at a higher risk of developing infective endocarditis. 5. Certain Conditions After Cardiac Transplantation: Cardiac transplant recipients who develop cardiac valvulopathy. It’s important to note that routine use of prophylactic antibiotics before dental procedures is not recommended for all patients with heart conditions. The decision should be made after considering the potential benefits and risks, and in consultation with the patient and their healthcare providers. In addition, the ADA and AHA guidelines recommend using the least number of doses necessary to reduce the risk of adverse drug reactions. A single dose of the antibiotic is typically administered 30 to 60 min before the procedure. For patients who are allergic to penicillin, alternative antibiotics are available. The choice of antibiotic should be guided by the patient’s medical history and potential drug interactions. Lastly, it’s important to note that effective oral hygiene and regular dental care are more effective in reducing the risk of bacteremia and subsequent infective endocarditis than the use of prophylactic antibiotics.”
	Hard	Which type of third molar impaction has the most pridctible outcome?	The mesioangular impaction, where the third molar is angled towards the front of the mouth, is often considered the most predictable type of third molar impaction. This type of impaction is relatively common and can be easier to extract compared to other types, such as horizontal or vertical impactions. However, the predictability of the outcome may also depend on individual factors, such as the position and development of the tooth, the skill of the dentist, and the patient’s overall oral health. It is important for dentists to carefully evaluate each case and discuss the potential risks and benefits with the patient before performing a third molar extraction.	“Third molar impaction is a common dental condition where the third molars, also known as wisdom teeth, do not have enough space to properly erupt or grow. The predictability of outcomes related to third molar impactions largely depends on the type of impaction. Impactions are typically classified based on the angle at which the impacted tooth is positioned relative to the second molar, and whether it’s fully or partially covered by bone or soft tissue. The four major types of impactions are: 1. Mesioangular impaction: The tooth is angled forward, towards the front of the mouth. 2. Distoangular impaction: The tooth is angled backward, towards the rear of the mouth. 3. Vertical impaction: The tooth is relatively upright but is unable to erupt due to lack of space. 4. Horizontal impaction: The tooth is lying horizontally and is perpendicular to the second molar. According to the American Association of Oral and Maxillofacial Surgeons (AAOMS) and the American Dental Association (ADA), the most predictable outcomes are generally seen with mesioangular impactions, especially those that are partially erupted. These are typically easier to remove than other types due to the angle and position of the tooth, and they often have fewer complications. Vertical impactions can also have predictable outcomes if they are not fully encased in bone, but the proximity to the second molar can sometimes make these extractions more challenging. Distoangular and horizontal impactions are often the most difficult to remove and may have a higher risk of complications, such as damage to the second molar, nerve injury, and dry socket. The predictability of outcomes also greatly depends on other factors such as the patient’s age, overall health, the density of the jawbone, root morphology, and the skill and experience of the surgeon. Therefore, a comprehensive evaluation and individualized treatment plan are essential for managing third molar impactions. Remember, routine removal of asymptomatic impacted wisdom teeth is not always necessary. The decision to remove or monitor should be made on a case-by-case basis, taking into consideration the potential benefits and risks of the procedure.”

**Table A8:** ChatGPT questions and answers about Implant.

Type	Difficulty	Question	ChatGPT 3.5	ChatGPT 4
Binary	Easy	Is minumm of 3 mm inter implant disatnce enough for placing dental implant?	The minimum inter-implant distance for placing dental implants may vary depending on the type of implant and the location in the mouth. However, in general, a minimum distance of 3 mm between implants is considered sufficient for proper placement and stability. This distance allows for adequate bone support and prevents potential complications, such as bone resorption and implant failure. Your dentist will carefully evaluate your case and determine the appropriate inter-implant distance based on your specific needs and treatment goals.	“The appropriate distance between dental implants is a critical factor to consider when planning for implant placement. It affects the health of the surrounding tissues, the distribution of forces, and the aesthetic result. According to the American Academy of Periodontology (AAP) and the Academy of Osseointegration (AO), a minimum distance of 3 mm between implants is typically recommended. This distance is believed to be necessary to maintain adequate bone and soft tissue health between the implants. The 3 mm distance is based on several factors: 1. Biologic considerations: Bone and soft tissue need adequate space for health and nutrition. A minimum distance of 3 mm allows for enough bone to remain between the implants, reducing the risk of bone loss and implant failure. 2. Prosthetic considerations: Adequate space between implants is necessary for proper contour and emergence profile of the final restoration, allowing for more natural-looking results. It also facilitates oral hygiene access. 3. Mechanical considerations: Adequate spacing helps distribute occlusal forces more evenly across the implants and the supporting bone, reducing the risk of overload and subsequent implant failure. While 3 mm is a general guideline, the exact spacing may need to be adjusted based on individual patient factors such as bone quality and quantity, the planned prosthesis, and the specific implant system being used. Therefore, careful planning, which may include diagnostic imaging and the use of surgical guides, is essential to ensure proper implant placement. Clinicians should also consider other factors such as the patient’s oral hygiene habits, overall health status, and aesthetic expectations when planning for implant placement. It should be noted that these guidelines may be updated as new research and clinical evidence become available. Clinicians should stay informed about the latest evidence and guidelines to ensure that their practice is based on the most current knowledge.”
	Medium	does dental impalnt surface texture/treatment affect treatment outcome?	Yes, dental implant surface texture and treatment can affect the treatment outcome. The surface texture of an implant refers to its microscopic roughness or smoothness, which can play a significant role in the osseointegration process (the fusion of the implant with the surrounding bone). Implants with a rough surface tend to have a higher success rate compared to smooth implants, as the rough surface allows for better adhesion and integration with the bone. Additionally, certain surface treatments, such as plasma spraying or acid etching, can further enhance the surface roughness and promote successful osseointegration. It is important for dentists to carefully select the appropriate implant surface texture and treatment for each individual case to ensure the best treatment outcome.	“Yes, the surface texture or treatment of a dental implant does indeed have an impact on treatment outcomes. The surface characteristics of dental implants can influence osseointegration, the process by which the dental implant becomes integrated with the surrounding bone. According to the American Academy of Periodontology (AAP) and the Academy of Osseointegration (AO), research has shown that surface-modified implants can enhance osseointegration and lead to higher success rates compared to machined or smooth surface implants. There are several methods used to modify the surface of dental implants, including: 1. Roughening: Techniques such as sandblasting, acid etching, or grit-blasting are used to increase the surface roughness of the implant. These rough surfaces promote bone cell attachment and proliferation. 2. Coating: Some implants are coated with materials such as hydroxyapatite or bioactive glass to promote bone growth. However, there has been some concern about the long-term stability of these coatings. 3. Disinfection: Some manufacturers provide implants that have undergone a final surface disinfection process to minimize the risk of bacterial contamination. 4. Surface Nano-technology: Some implants are designed with nano-scale topographies that can enhance cellular responses and speed up the osseointegration process. It’s important to note that while surface modifications can enhance osseointegration, they are just one of many factors that contribute to the success of dental implant therapy. Other factors, such as the patient’s health status, bone quality and quantity, oral hygiene habits, surgical technique, and implant design, also play significant roles. Clinicians should consider all these factors when planning for dental implant therapy, and keep up-to-date with the latest research and guidelines to ensure they are providing the best possible care for their patients.”
	Hard	Is there contraindications for placing dental implants?	“Yes, there are some contraindications for placing dental implants, including: 1. Insufficient bone: Adequate bone support is necessary for successful implant placement and osseointegration. Patients with severe bone loss or atrophy may not be suitable candidates for dental implants. 2. Uncontrolled systemic diseases: Certain medical conditions, such as uncontrolled diabetes or autoimmune disorders, can affect the body’s ability to heal and increase the risk of complications during implant surgery. 3. Smoking: Smoking can impair the body’s healing process and increase the risk of implant failure. 4. Poor oral hygiene: Good oral hygiene is essential for the success of dental implants. Patients with a history of poor oral hygiene may not be able to maintain the necessary level of cleanliness around the implant, leading to potential complications. It is important for patients to discuss their medical history and any concerns with their dentist before considering dental implant treatment.”	“Yes, there are certain conditions that might contraindicate the placement of dental implants. According to the American Academy of Periodontology (AAP) and the Academy of Osseointegration (AO), these include: 1. Uncontrolled Systemic Diseases: Certain systemic diseases, such as uncontrolled diabetes, conditions affecting bone turnover (e.g., osteoporosis treated with bisphosphonates), immune deficiencies, or other diseases that could adversely affect healing, may contraindicate implant placement or require additional consideration and consultation with the patient’s physician. 2. Active Infection: Active infection in the area where the implant is to be placed, such as periapical pathology or periodontal disease, should be resolved prior to implant placement. 3. Insufficient Bone: If the patient lacks sufficient bone to support an implant, bone augmentation procedures might be required. However, in some cases, if the required augmentation is extensive, it might be a contraindication. 4. Poor Oral Hygiene: Poor oral hygiene can increase the risk of peri-implant diseases, including peri-implant mucositis and peri-implantitis. 5. Heavy Smokers: Smoking is associated with a higher risk of dental implant failure and complications. 6. Substance Abuse: Alcohol or drug abuse can negatively influence oral health and the success of dental implants. 7. Psychiatric Illness: Certain psychiatric conditions or a lack of mental readiness for the procedure and aftercare can contraindicate implant placement. 8. Radiation Therapy: Patients who have received high-dose radiation treatment to the head or neck may have an increased risk of implant failure. 9. Growth and Development: Implants should generally not be placed in individuals who are not fully grown. 10. Pregnancy: Due to changes in hormone levels and possible discomfort, it’s typically recommended to avoid implant surgery during pregnancy. It’s important to note that these contraindications can be relative and vary from patient to patient. The decision to place implants should be made after a thorough examination and consultation, considering the patient’s overall health status, oral health situation, and their preferences. The risks and benefits of the treatment should be explained, and in some cases, consultation with the patient’s physician may be necessary.”
Descriptive	Easy	Which area of the mouth has the best quality and quanitiy of bone for placing dental implant?	The area with the best quality and quantity of bone for placing dental implants is the mandible, specifically the posterior region near the mental foramen. This area has a higher bone density compared to the maxilla (upper jaw) and is less prone to bone resorption. Additionally, the bone in this area is typically thicker, providing better support and stability for the implant. However, the amount and quality of bone can vary from person to person, and it is important for dentists to carefully evaluate each case to determine the best location for implant placement.	“The quantity and quality of bone available for dental implant placement are important factors that can influence the success of the procedure. According to the Academy of Osseointegration (AO) and the American Academy of Periodontology (AAP), certain areas of the mouth tend to have better bone characteristics for dental implants. In general, the anterior region of the lower jaw (mandible), specifically the area around the canines and premolars, often has the greatest bone density and volume, making it a favorable site for implant placement. The bone in this region is typically dense, and the area is well-protected from the forces of mastication, which makes it a good site for osseointegration. The posterior region of the lower jaw also has good bone quality, but the quantity may be limited due to the proximity of the inferior alveolar nerve, which should be avoided during implant placement. The anterior region of the upper jaw (maxilla) generally has less bone density compared to the lower jaw, but there is often a good quantity of bone available for implant placement. However, the maxillary sinus may limit the bone volume in the posterior region of the upper jaw. Sinus lift or sinus augmentation procedures can be performed to increase the bone volume in this area if necessary. It’s important to note that individual variations can be significant, and the quantity and quality of bone can be affected by factors such as periodontal disease, tooth loss, trauma, and other conditions. A thorough examination, including radiographic assessment, should be conducted to evaluate the bone characteristics in each individual patient. In cases where there is insufficient bone for implant placement, various bone augmentation procedures can be performed to increase the bone volume. The decision to perform these procedures should be based on a comprehensive evaluation of the patient’s condition and their treatment goals.”
	Medium	How can we reduce bone overheating while placing dental implant?	“To reduce bone overheating while placing dental implants, dentists may use a variety of techniques and instruments, such as: 1. Cooling irrigation: Constantly irrigating the surgical site with cool saline or water can help dissipate heat and prevent bone overheating. 2. Low-speed drilling: Using a low-speed drill can generate less heat compared to high-speed drills, reducing the risk of bone overheating. 3. Proper drilling technique: Proper technique, including intermittent drilling and constant irrigation, can help prevent excessive heat buildup. 4. Using sharp drills: Dull drills can generate more heat due to increased friction, so it is important for dentists to use sharp, well-maintained drills. 5. Monitoring temperature: Some implant systems come with a temperature monitoring device that can alert the dentist if the bone temperature rises above a certain threshold. It is important for dentists to carefully follow these techniques and precautions to ensure successful implant placement and minimize potential complications.”	“Avoiding overheating of the bone during dental implant placement is crucial, as excessive heat can cause bone necrosis and negatively impact the success of the procedure. The American Academy of Periodontology (AAP) and the Academy of Osseointegration (AO) provide several strategies to prevent overheating: 1. Use Sharp Drills: Always use sharp drills when preparing the implant site. Dull drills can increase friction and generate heat. 2. Low-Speed Drilling: Use low-speed drilling with high torque to minimize heat production. The recommended speed may vary based on the manufacturer’s instructions and the specific stage of the osteotomy, but speeds less than 1000 rpm are often recommended. 3. Intermittent Drilling: Use an intermittent drilling technique, rather than continuous drilling. This allows the bone to cool down between drilling intervals. 4. Adequate Irrigation: Use copious external and internal irrigation with sterile saline solution during drilling to cool the drill and the bone. Some implant systems also have drills designed to improve the flow of the coolant. 5. Gradual Drilling: Incrementally increase the size of the drills used to prepare the implant site. This reduces the amount of bone removed in each step, which can help to reduce heat generation. 6. Bone Quality: Consider the quality of the bone. Denser bone may require more irrigation and slower drilling speeds to avoid overheating. 7. Drill Maintenance: Regularly maintain and replace drills as per the manufacturer’s instructions to ensure optimal performance. 8. Patient Factors: Be aware of patient factors that might affect bone healing, such as smoking, certain medications, and systemic diseases. By carefully considering these factors and following recommended protocols, clinicians can minimize the risk of bone overheating during implant placement. It’s important to stay updated with the latest guidelines and research to ensure the best possible outcomes for patients.”
	Hard	What techinques can be used for assessing dental implant stability?	“Some techniques for assessing dental implant stability may include: 1. Periotest: This device measures the mobility of the implant by tapping it with a small probe and recording the results on a numerical scale. A stable implant will have a lower Periotest value. 2. Resonance Frequency Analysis (RFA): This technique uses a small device to measure the implant’s stability and bone-to-implant contact by vibrating the implant and recording its frequency response. 3. Radiographs: X-rays can be used to evaluate the implant’s position and stability in relation to the surrounding bone. 4. Reverse torque testing: This method involves applying force to the implant in the opposite direction of its placement to assess its stability and resistance to movement. 5. Clinical observation: Dentists may also use their clinical experience and judgment to assess implant stability, including evaluating the implant’s mobility and checking for any signs of inflammation or infection around the implant. It is important for dentists to use a combination of these techniques to accurately assess dental implant stability.”	The stability of a dental implant is a critical factor that influences its success. Various methods have been developed to assess implant stability. According to the American Academy of Periodontology (AAP) and the Academy of Osseointegration (AO), these include: 1. Periotest: This device measures the damping characteristics of the bone-implant interface, providing a quantitative assessment of implant stability. 2. Resonance Frequency Analysis (RFA): This is a non-invasive method that uses the concept of resonance frequency to measure the stiffness of the bone-implant interface. The most commonly used device for RFA is the Osstell ISQ, which provides an Implant Stability Quotient (ISQ) value. 3. Computerized Tomography (CT) or Cone Beam Computed Tomography (CBCT): These imaging techniques can provide detailed information about the quality and quantity of the surrounding bone, and can help assess the integration of the implant with the bone. 4. Surgical Assessment: During the placement of the implant, the clinician can assess the quality of the bone and the initial stability of the implant. 5. Mobility Testing: This is a manual method where the clinician uses a dental instrument to apply small forces to the implant to assess its mobility. However, this method is generally subjective and is used when other methods suggest a problem. 6. Histological Analysis: While not used in routine clinical practice, histological analysis of bone samples obtained during surgery can provide detailed information about the bone-implant interface. It’s important to note that each of these methods has its advantages and limitations. The choice of method can depend on various factors, including the specific clinical situation, the timing of the assessment (e.g., immediate post-placement, during healing, or after loading), and the clinician’s experience and preference. Regular assessment of implant stability can help identify potential problems early and allow for interventions to improve the outcome. However, the assessment of implant stability is just one aspect of the comprehensive evaluation and monitoring of dental implant treatment. A successful outcome also depends on various other factors, including patient health, surgical technique, oral hygiene, and prosthesis design.
